# Metal-bio functionalized bismuthmagnetite [Fe_3−*x*_Bi_*x*_O_4_/SiO_2_@l-ArgEt_3_^+^I^−^/Zn(ii)]: a novel bionanocomposite for the synthesis of 1,2,4,5-tetrahydro-2,4-dioxobenzo[*b*][1,4]diazepine malononitriles and malonamides at room temperature and under sonication†

**DOI:** 10.1039/d2ra00212d

**Published:** 2022-04-01

**Authors:** Fatemeh Molaei Yielzoleh, Kobra Nikoofar

**Affiliations:** Department of Chemistry, Faculty of Physics and Chemistry, Alzahra University Tehran Iran k.nikoofar@alzahra.ac.ir kobranikoofar@yahoo.com

## Abstract

In this work, a new magnetized composite of bismuth (Fe_3−*x*_Bi_*x*_O_4_) was prepared and functionalized stepwise with silica, triethylargininium iodide ionic liquid, and Zn(ii) to prepare a multi-layered core–shell bio-nanostructure, [Fe_3−*x*_Bi_*x*_O_4_/SiO_2_@l-ArgEt_3_^+^I^−^/Zn(ii)]. The modified bismuth magnetic amino acid-containing nanocomposite was characterized using several techniques including Fourier-transform infrared (FT-IR), X-ray fluorescence (XRF), vibrating sample magnetometer (VSM), field-emission scanning electron microscopy (FESEM), energy dispersive X-ray analysis (EDAX), thermogravimetric/differential scanning calorimetric (TGA/DSC) analysis, X-ray photoelectron spectroscopy (XPS), Brunauer–Emmett–Teller (BET), and inductively coupled plasma-optical emission spectrometry (ICP-OES). The magnetized bionanocomposite exhibited high catalytic activity for the synthesis of 1,2,4,5-tetrahydro-2,4-dioxobenzo[*b*][1,4]diazepine malononitriles *via* five-component reactions between 1,2-phenylenediamines, Meldrum's acid, malononitrile, aldehydes, and isocyanides at room temperature in ethanol. The efficacy of this protocol was also examined to obtain malonamide derivatives *via* pseudo six-component reactions of 1,4-phenylenediamine, Meldrum's acid, malononitrile, aldehydes, and isocyanides. When the above-mentioned MCRs were repeated under the same conditions with the application of sonication, a notable decrease in the reaction time was observed. The recovery and reusability of the metal-bio functionalized bismuthmagnetite were examined successfully in 3 runs. Furthermore, the characteristics of the recovered Fe_3−*x*_Bi_*x*_O_4_/SiO_2_@l-ArgEt_3_^+^I^−^/Zn(ii) were investigated though FESEM and EDAX analysis.

## Abstract 2

In this work, a new magnetized composite of bismuth (Fe_3−*x*_Bi_*x*_O_4_) was prepared and functionalized stepwise with silica, triethylargininium iodide ionic liquid, and Zn(ii) to prepare a multi-layered core–shell bio-nanostructure, [Fe_3−*x*_Bi_*x*_O_4_/SiO_2_@l-ArgEt_3_^+^I^−^/Zn(ii)].

## Graphical abstract

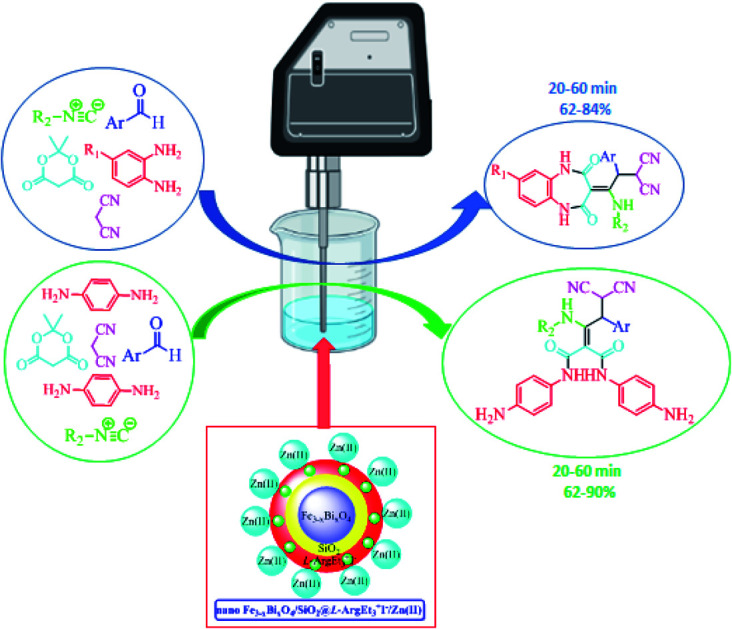

## Introduction

Multi-component reactions (MCRs) are versatile strategies in organic synthesis, which utilize more than two different starting substances to prepare a single product. The first MCR was accomplished by Strecker in 1850, resulting in the formation of α-amino nitriles. Subsequently, the role of MCRs in the preparation of various classes of organic compounds increased rapidly. In fact, MCRs, as smart types of transformations resulted in the evolution of organic synthesis and sustainable chemistry.^1–3^ MCRs are also versatile tools in drug discovery and medicinal chemistry,^4^ asymmetric synthesis,^5^ preparation of bioactive molecules,^6^ ligation and bioconjugation chemistry,^7^ synthesis of polyheterocycles and complex molecules,^8^ peptide macrocyclization and stapling,^9^ and steroid diversification.^10^ Another remarkable feature of MCRs is that the combination of more than two MCRs, which leads to a union of MCRs, multiplies their efficacy.^11^ In fact, the occurrence of union MCRs increases the possibility of the one-pot synthesis of more substrates to obtain a single complex product in an economical and environment-friendly procedure.

The preeminent efficacy of MCRs in comparison to stepwise reactions is due to some notable points such as their simple operation *via* a one-pot reaction, atom-economy, high chemoselectivity and/or stereoselectivity, reduced energy consumption, waste reduction by avoiding the purification and separation of intermediates, short procedures, slight protection and deprotection of functional groups, and high yields of the main product without the formation of by-products.^12^

Benzodiazpines (BDZs) are important bicyclic aromatic heterocyclic scaffolds made up of a benzene ring fused to a seven-membered ring containing two nitrogen atoms. Based on the positions of the nitrogen atoms, many types of benzodiazpines exist such as 1,2-BDZs, 1,3-BDZs, 1,4-BDZs, 1,5-BDZs, and 2,3-BDZs.^13^ Various classes of organics and functionalized heterocycles, which include diverse types of BDZ motifs, play critical role in medicinal chemistry, pharmacology, and treatment.^14^ BDZ is a key synthon in different therapeutic chemicals and drugs, which possess a wide range of properties such as antiarrhythmic,^15^ antidepressant,^16^ anticonvulsant,^17^ analgesic,^18^ and antituberculosis.^19^ Alprazolam (a short-acting tranquilizer), bromazepam (for anxiety treatment), brotizolam (used for short-term treatment of severe insomnia), and lorazepam (used to treat anxiety disorders and sleeping troubles) are some drugs that possess a benzodiazepine skeleton.

Malonamide derivatives (MDs) have gained significant attention due to their interesting properties in various fields of science and technology.^20^ MDs are important organics in the fields of medicinal chemistry, drugs, and biology as antiinflammatories,^21^ potent agonists of TGR5 (which are promising molecular targets for metabolic diseases),^22^ selective κ opioid receptor agonists,^23^ potent α-glucosidase inhibitors,^24^ γ-secretase inhibitors for the potential treatment of Alzheimer's disease,^25^ and antibiotics against methicillin-resistant *Staphylococcus aureus*.^26^ Various classes of compounds bearing malonamide building blocks act as gelators,^27^ extractors in separation technology,^28^ materials for the recovery of rare-earth metals from end-of-life products (lamp phosphors),^29^ and stabilizers for nitrocellulose-based propellants.^30^

Magnetized nanostructures can be prepared *via* the metal doping of magnetite with the general formula of Fe_3−*x*_M_*x*_O_4_ (M = Ni, Zn, Mg, Co, Mg, Ta, *etc.*) employing various methods. In 1998, a crystalline film of spinel Fe_3−*x*_M_*x*_O_4_ (M = Ni and Zn) was prepared through a spine-spray ferrite plating method and its crystallographic and magnetic properties were studied.^31*a*^ The Opel group reported thin films of the ferrimagnetic spinel oxide Zn_*x*_Fe_3−*x*_O_4_ as spintronic materials with tunable electrical and magnetic properties.^31*b*^ The Alrozi group, in 2019, utilized an Fe_3−*x*_Mn_*x*_O_4_–MKSF composite catalyst for the degradation of acid orange II dye thorough a heterogeneous Fenton-like reaction.^31*c*^ The He group, in 2013, studied the valence and site occupancy of substituted metals in the magnetite spinel structure Fe_3−*x*_M_*x*_O_4_ (M = Cr, Mn, Co, and Ni) and their influence on its thermal stability.^31*d*^ The Gusevskaya research group, in 2004, reported the oxidation of β-pinene in the presence of Fe_3−*x*_M_*x*_O_4_ (M = Co and Mn) as heterogenous catalysts.^31*e*^ He's group, in 2009, examined the decolorization of methylene blue *via* a heterogeneous Fenton reaction using Fe_3−*x*_Ti_*x*_O_4_ at neutral pH.^32^ Some catalytic systems also have been applied based on a titanomagnetite (Fe_3−*x*_Ti_*x*_O_4_) core to promote organic transformations.^33^ The Walz group, in 2002, studied some characteristics of Fe_3−*x*_M_*x*_O_4_ (M = Ni, Mg, Co, Al, Ti, and Ga) *via* magnetic after-effect (MAE) spectroscopy.^34^ However, although the investigations on Fe_3−*x*_M_*x*_O_4_ with different metals are comprehensive, in the literature, there are no reports on Fe_3−*x*_Bi_*x*_O_4_. Sun's group, in 2019, prepared and characterized bismuth-doped Ni–Cu–Co nano ferrites (Ni_0.2_Cu_0.2_Co_0.6_Fe_2−*x*_Bi_*x*_O_4_) *via* sol–gel auto-combustion technology.^35^ Praveena *et al.*, in 2014, synthesized NiFe_2−*x*_Bi_*x*_O_4_ nanopowder *via* a chemical co-precipitation method.^36^

It should be mentioned that some composites have been prepared by loading bismuth on magnetite. For example, Cai's research group, in 2017, prepared a Bi/Fe_3_O_4_ composite through a one-pot process from ferrous sulfate and bismuth chloride using hydrazine hydrate as a reducing agent. They examined its catalytic performances for 4-nitrophenol reduction.^37*a*^ Hasanpour's group, in 2013, synthesized a Bi–Fe_3_O_4_ nanocomposite mechanochemically and investigated its dielectric behavior.^37*b*^ Gao and coworkers, in 2015, loaded bismuth and Fe_3_O_4_ nanoparticles on reduced graphene oxide to fabricate Bi–Fe_3_O_4_@RGO hybrids, which catalyzed the reduction of 4-nitrophenol.^37*c*^

Silica-coated magnetized nanostructures have attracted increasing attention in multiple fields of science and technology.^38^ Pratapa's group, in 2019, prepared amorphous-silica-coated magnetite-nanoparticle (Fe_3_O_4_/α-SiO_2_) composites *via* co-precipitation and modified Stöber methods, respectively.^39*a*^ These nanocomposites have been utilized in various aspects of applied technologies such as dye removal as sorbents,^39*b*^ carriers of hydrophilic polymer as high-density polymer brush shell,^39*c*^ protein isolation,^39*d*^ antimicrobial agents,^39*e*^ rewarming organs through nanowarming technology,^39*f*^ and bifunctional agents for magnetic resonance imaging and Zn(ii) fluorescent sensing.^39*g*^

Recently, promoting different MCRs in the presence of various catalytic systems containing silica-coated magnetized cores has gained considerable interest. In 2017 Mobinikhaledi's group reported the use of sodium polyaspartate-functionalized silica-coated magnetite nanoparticles (MNPs-SPAsp) as heterogeneous and reusable catalysts for the solvent-free synthesis of 2-amino-4*H*-chromenes.^40*a*^ Shirini *et al.*, in 2020, utilized silica-coated magnetic nanoparticles containing bis dicationic bridge (γ-Fe_2_O_3_@SiO_2_@[Bis-APTES]Cl_2_) for the synthesis of 1,2,4-triazolo pyrimidine/quinazolinone derivatives.^40*b*^ In 2021, Baharfar prepared indol-3-yl-4*H*-chromenes in the presence of Fe_3_O_4_@SiO_2_@D-NHCS-Tr under solvent-free conditions.^40*c*^ Mao's group immobilized phospholipase D on silica-coated magnetic nanoparticles to obtain functional phosphatidylserines.^40*d*^ In 2021, Gholizadeh and coworkers applied copper-functionalized silica-coated magnetic nanoparticles for an efficient Suzuki cross-coupling reaction.^40*e*^

Currently, bio-based nanostructures and nanocomposites are important in various scientific and technological fields. They possess broad-range of applications in medicine, packaging, consumer goods, electronics, transportation, construction, and green processes.^41*a*^ The antimicrobial, industrial, and biomedical applications of gum bio-based nanocomposites were reported in 2019.^41*b*^ Edible bio-based nanostructures (as nanocarriers of bioactive compounds to specific body sites) and their delivery, absorption and potential toxicity were also previously discussed.^41*c*^ Some nanostructured bio-based carbon electrodes were exploited for energy storage applications.^41*d*^ They were also utilized in cancer therapy and oncotherapy due to their potent properties such as autologous pharmaceutical and synergetic effects, biocompatibility, biodegradability and biosafety.^41*e*^

Amino acids are a great and attractive class of bio-based compounds, which play a critical role in organic and green chemistry. Many types of catalytic systems such as amino acidic-based ionic liquids (AAILs) and amino acid-containing bionanocomposites have been reported to promote various types of MCRs and other applications in science and technology.^42^

Arginine (Arg), a charged aliphatic amino acid at physiological pH, is a semi-essential α-amino acid utilized in the biosynthesis of proteins. Thus, arginine and arginine-containing structures are significant in variant application fields. A vast range of ligands and composites possessing Arg motifs are used as surfactants,^43^ sticky protein equivalents for viable cell accommodation,^44^ and membranes for carbon capture^45^ and ion removal from water.^46^

Green chemistry is a valuable environmentally friendly concept in many branches of science and technology. Based on the 12 principles introduced and explained by Anastas and Warner in 1998 for the first time, the enlarged “green chemisTREE” was introduced in 2018 and the concept of “periodic table of green and sustainable chemistry” was recommended in 2019.^47^ Nowadays, due to the critical role of green chemistry, different research, data, and discoveries form various scientific and operational perspectives such as green techniques to perform reactions^48^ utilizing green catalysts and reagents in chemical transformations, drug delivery, and medicine,^49^ and solvent-free or green-media reactions.^50^

**Scheme 1:**
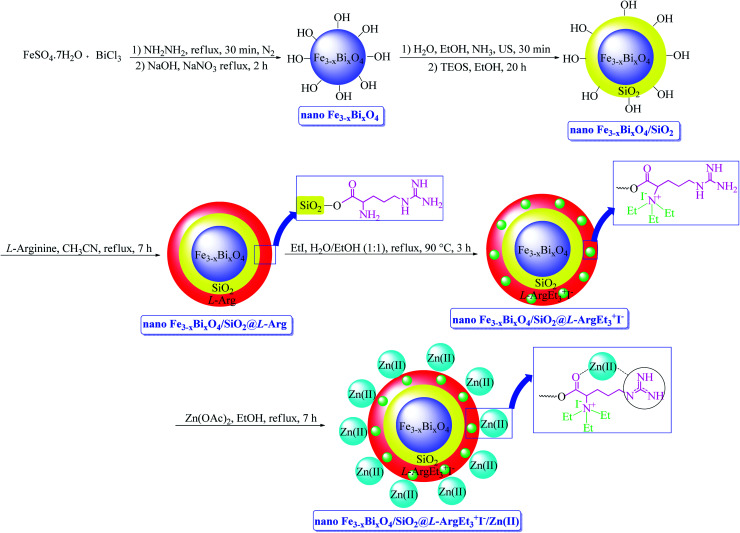
Synthesis of Fe_3−*x*_Bi_*x*_O_4_/SiO_2_@l-ArgEt_3_^+^I^−^/Zn(ii) bionanocomposite.

**Scheme 2:**
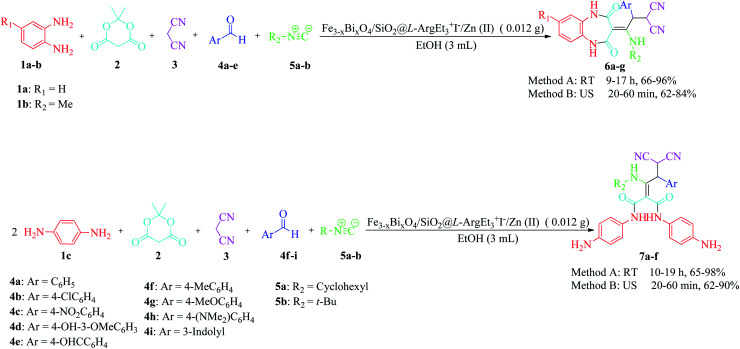
Synthesis of 4,5-tetrahydro-2,4-dioxobenzo[*b*][1,4]diazepine malononitriles 6a–g and malonamides 7a–f in the presence of nano Fe_3−*x*_Bi_*x*_O_4_/SiO_2_@l-ArgEt_3_^+^I^−^/Zn(ii).

In continuation of our interest in preparing novel magnetized nanostructures to promote MCRs under green conditions,^51^ herein, we report the preparation of a novel magnetized multi-layered core–shell bionanostructure, [Fe_3−*x*_Bi_*x*_O_4_/SiO_2_@l-ArgEt_3_^+^I^−^/Zn(ii)]. The organic–inorganic bio-hybrid obtained forms the Fe_3−*x*_Bi_*x*_O_4_ core, which was functionalized stepwise through the immobilization of silica, triethylargininium iodide ionic liquid, and Zn(ii) (Scheme 1). The synthesized nanostructure was examined as a heterogenous catalyst for the synthesis of 2,4,5-tetrahydro-2,4-dioxobenzo[*b*][1,4]diazepine malononitriles and malonamides in ethanol at room temperature (method A) and also in the presence of ultrasound irradiation (method B) (Scheme 2).

## Results and discussion

### Characterization of the bionanocomposite

**Fig. 1:**
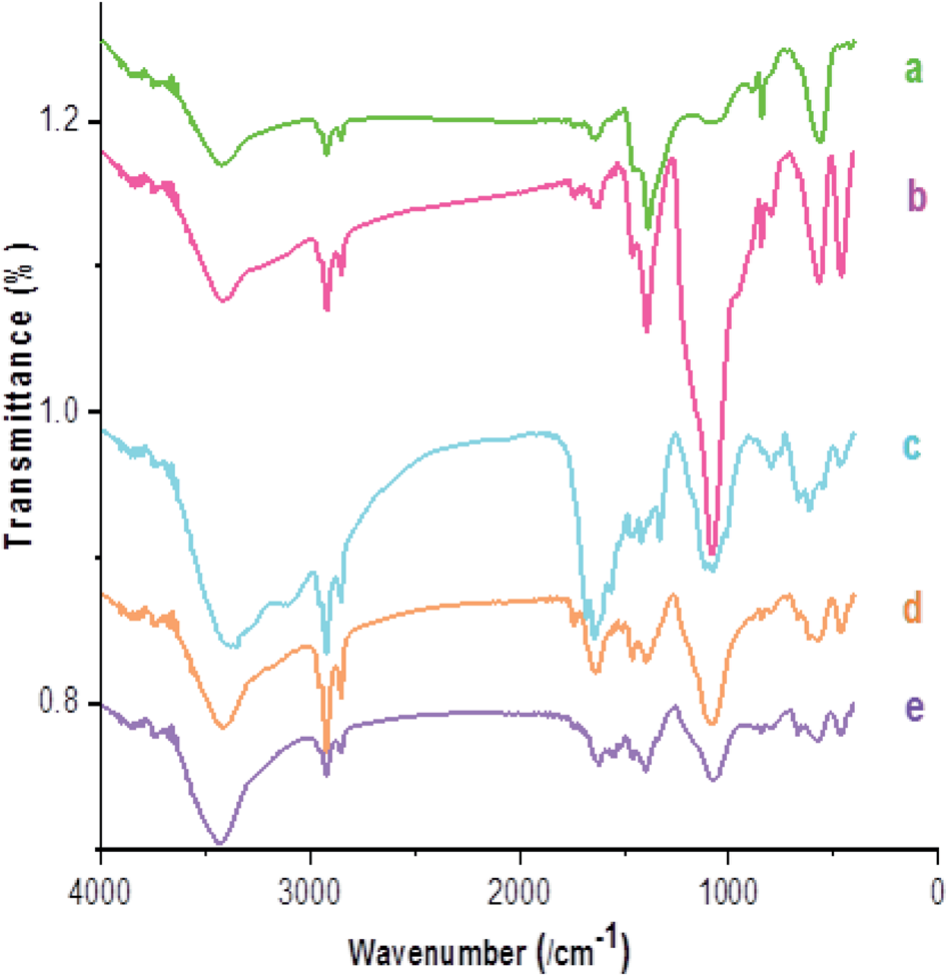
FT-IR spectra of (a) Fe_3−*x*_Bi_*x*_O_4_, (b) Fe_3−*x*_Bi_*x*_O_4_/SiO_2_, (c) Fe_3−*x*_Bi_*x*_O_4_/SiO_2_@l-Arg, (d) Fe_3−*x*_Bi_*x*_O_4_/SiO_2_@l-ArgEt_3_^+^I^−^, and (e) Fe_3−*x*_Bi_*x*_O_4_/SiO_2_@l-ArgEt_3_^+^I^−^/Zn(ii).

The FT-IR spectra of Fe_3−*x*_Bi_*x*_O_4_ (a), Fe_3−*x*_Bi_*x*_O_4_/SiO_2_ (b), Fe_3−*x*_Bi_*x*_O_4_/SiO_2_@l-Arg (c), Fe_3−*x*_Bi_*x*_O_4_/SiO_2_@l-ArgEt_3_^+^I^−^ (d), and Fe_3−*x*_Bi_*x*_O_4_/SiO_2_@l-ArgEt_3_^+^I^−^/Zn(ii) (e) are presented in Fig. 1. According to Fig. 1a, the peak at 563 cm^−1^ and 844 cm^−1^ are attributed to the stretching vibration of the Fe–O bond and symmetrical stretching vibration of the Bi–O bonds, respectively. The very weak peak at 1047 cm^−1^ can be ascribed to some other vibration of Bi–O caused through the interaction between the Bi–O bonds and their surroundings. The broad band at 3440 cm^−1^ and the peak at 1633 cm^−1^ are related to the stretching and bending vibrations of the hydroxyl group, respectively.^52^ As shown in Fig. 1b, the appearance of bands at 1085 cm^−1^ (Si–O–Si stretching vibrations), 790 cm^−1^ (Si–O–Si bending vibrations), and 466 cm^−1^ (Si–O–Si rocking vibrations) confirmed the embedding of silica on the bismuthmagnetite core.^53^ The peaks at 3253 cm^−1^ and 3122 cm^−1^ (stretching vibration of NH and NH_2_ bonds), 1647 cm^−1^ (bending vibration of N–H), 1679 cm^−1^ (C=O stretching vibration), and 1112 cm^−1^ (C–N stretching vibration), as shown in Fig. 1c, confirmed the presence of l-Arginine on the surface of Fe_3−*x*_Bi_*x*_O_4_/SiO_2_@l-Arg. As shown in Fig. 1d, the weakening of the bands in the region of the N–H stretching vibrations represented the settling of EtI on the structure. According to Fig. 1e, the shifting the peaks of the C=O stretching vibrations form 1679 cm^−1^ to 1656 cm^−1^ confirmed the coordination of Zn(ii) to l-ArgEt_3_^+^I^−^, as the outer layer of the bionanocomposite.

**Fig. 2:**
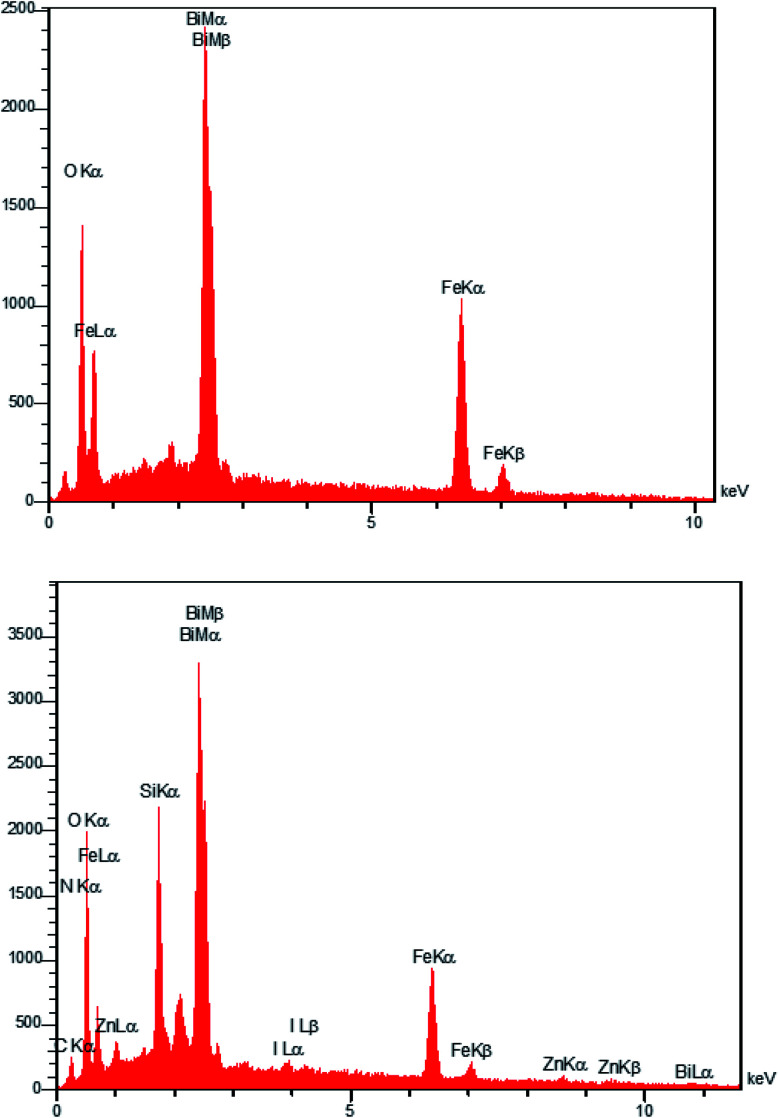
EDAX analysis of Fe_3−*x*_Bi_*x*_O_4_ (top) and Fe_3−*x*_Bi_*x*_O_4_/SiO_2_@l-ArgEt_3_^+^I^−^/Zn(ii) (bottom).

The EDAX analysis of the Fe_3−*x*_Bi_*x*_O_4_ core in Fig. 2 (top) revealed the presence of iron (29.96 wt%), bismuth (41.66 wt%), and oxygen (28.38 wt%). No other impurities based on the compounds utilized during the preparation procedure were observed. The bottom diagram in Fig. 2 confirms that Fe_3−*x*_Bi_*x*_O_4_/SiO_2_@l-ArgEt_3_^+^I^−^/Zn(ii) is made up of iron (17.02 wt%), bismuth (39.84 wt%), oxygen (26.16 wt%), silicon (6.05 wt%), carbon (7.08 wt%), nitrogen (0.31 wt%), iodine (1.34 wt%), and zinc (2.20 wt%). These results confirm the successful preparation of the bio multi-layered Fe_3−*x*_Bi_*x*_O_4_/SiO_2_@l-ArgEt_3_^+^I^−^/Zn(ii) nanostructure. In addition, the ICP-OES analysis demonstrated that the catalyst contains 30 963.3 ppm of Zn (3.096% Zn or 0.473 mmol Zn per 1 g of catalyst).

**Fig. 3:**
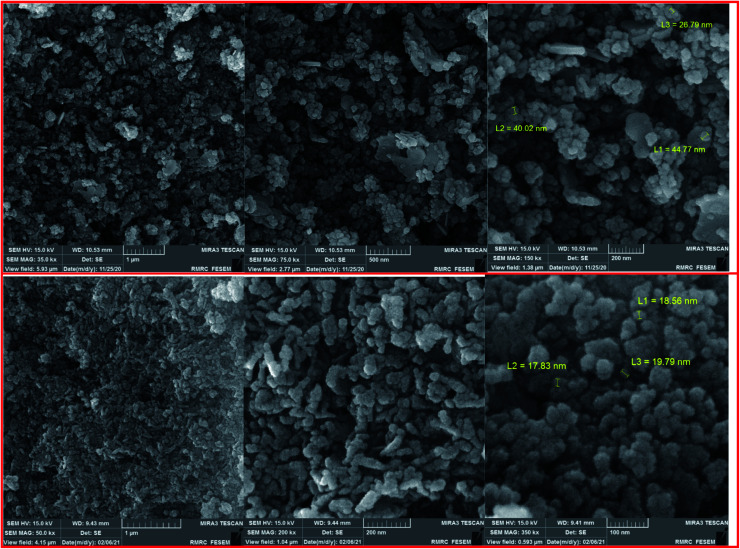
FESEM images of Fe_3−*x*_Bi_*x*_O_4_ (top) and Fe_3−*x*_Bi_*x*_O_4_/SiO_2_@l-ArgEt_3_^+^I^−^/Zn(ii) (bottom).

The FESEM images of the Fe_3−*x*_Bi_*x*_O_4_ core (top) and the final magnetized bionanocomposite Fe_3−*x*_Bi_*x*_O_4_/SiO_2_@l-ArgEt_3_^+^I^−^/Zn(ii) (bottom) are illustrated in Fig. 3. According to the top images, the core structure exhibited semi-uniform nano-sized particles with an average size of 25–45 nm. The final nanostructure consisted of more uniform semi-spherical and pseudo-filamentary nano-sized particles with an average size of 15–20 nm. Generally, complete uniformity was not observed in the morphology of the nanostructures throughout the bismuthmagnetite core and the final bionanocomposite.

The XRF analysis of the Fe_3−*x*_Bi_*x*_O_4_ core exhibited the presence of Fe_2_O_3_ (16.394%) and Bi (82.565%), confirming the successful synthesis of bismuthmagnetite (Table 1).

**Table 1:** XRF analysis of Fe_3−*x*_Bi_*x*_O_4_ core

	Fe_2_O_3_	Bi	LOI
(%)	16.394	82.565	0.2953

**Fig. 4:**
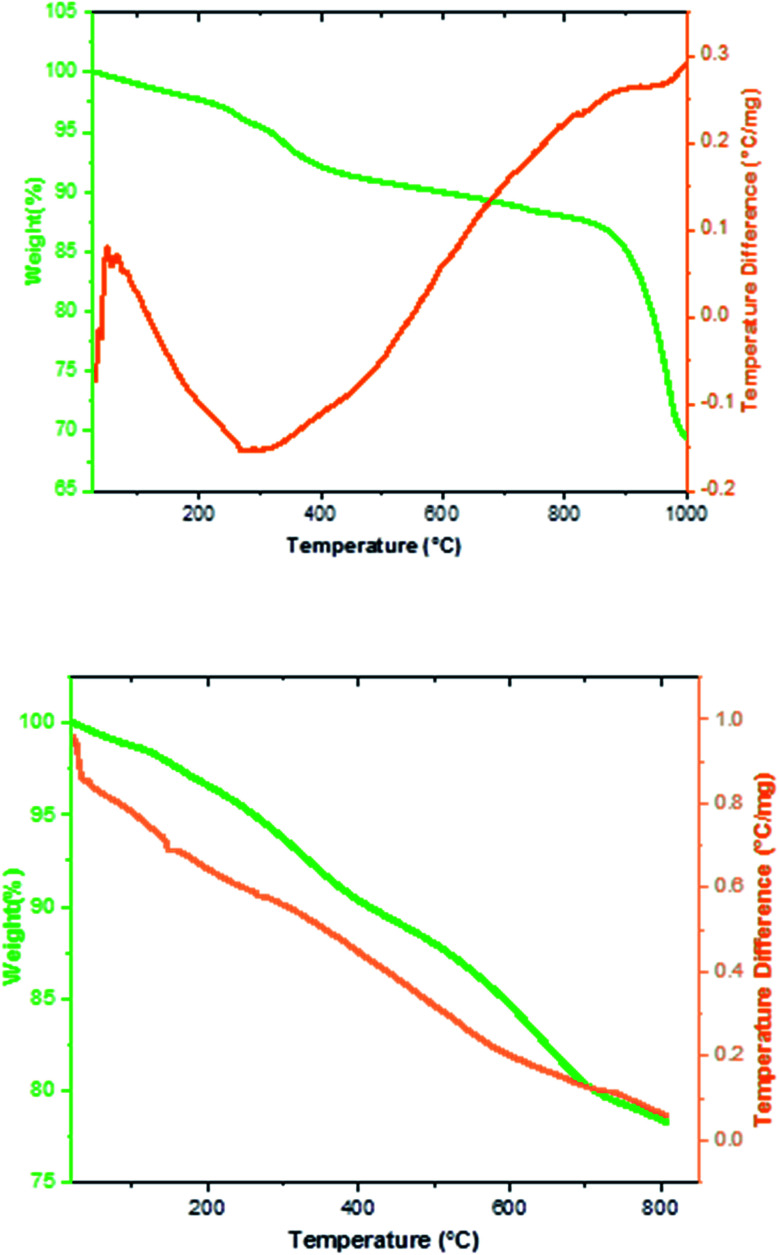
TGA/DSC curves of Fe_3−*x*_Bi_*x*_O_4_ (top) and Fe_3−*x*_Bi_*x*_O_4_/SiO_2_@l-ArgEt_3_^+^I^−^/Zn(ii) (bottom).

The thermal behavior of the Fe_3−*x*_Bi_*x*_O_4_ MNPs and Fe_3−*x*_Bi_*x*_O_4_/SiO_2_@l-ArgEt_3_^+^I^−^/Zn(ii) nanocomposite was investigated *via* TGA/DSC up to 1000 °C, as shown in Fig. 4. Based on the top diagram, which relates to the Fe_3−*x*_Bi_*x*_O_4_ core, the initial endothermic weight loss occurred at 260–400 °C (8%). The second weight loss of about 13.3% was observed at 830–870 °C. Heating the Fe_3−*x*_Bi_*x*_O_4_ core up to 1000 °C yielded the endothermic total weight loss of 31.38%. Thus, the core is almost completely thermally stable up to 400 °C. The TGA/DSC curves of Fe_3−*x*_Bi_*x*_O_4_/SiO_2_@l-ArgEt_3_^+^I^−^/Zn(ii) in bottom diagram of Fig. 4 demonstrate the first mass loss of about 2.2% at 150 °C. The second mass loss observed at 267 °C was about 5.2%, corresponding to the decomposition of the outer layers of the bionanocomposite, which are organic compounds (the l-ArgEt_3_^+^I^−^ IL). At 710 °C the total wight loss of about 20% was observed, and at 800 °C, the total weight loss was 21.8%. All the steps are endothermic and the wight changes occurred continuously with a mild decrease.

**Fig. 5:**
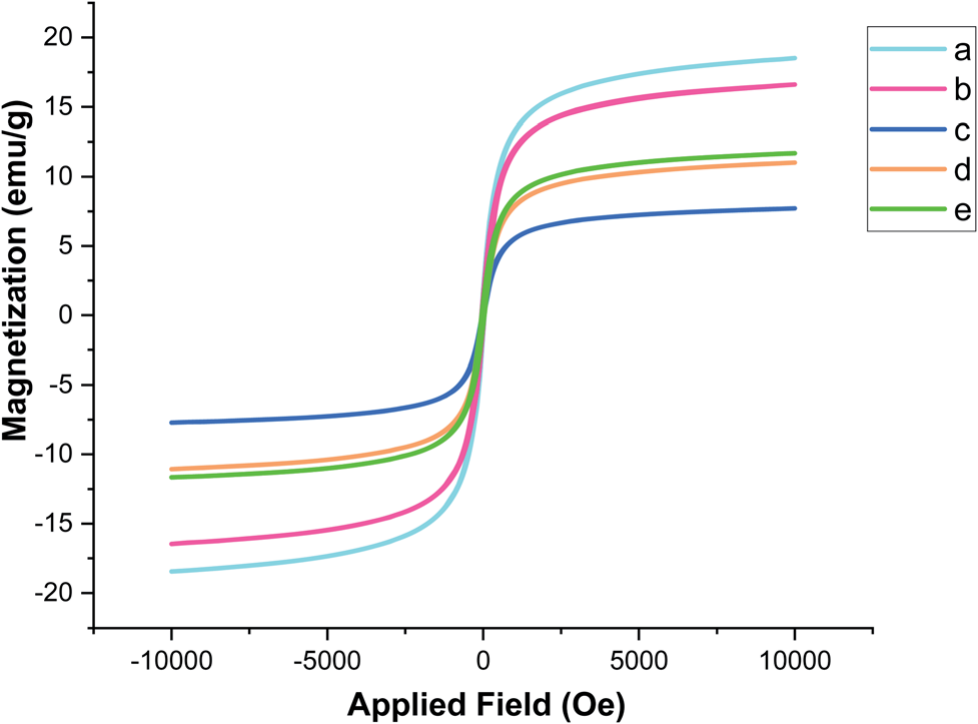
Magnetization curves of (a) Fe_3−*x*_Bi_*x*_O_4_, (b) Fe_3−*x*_Bi_*x*_O_4_/SiO_2_, (c) Fe_3−*x*_Bi_*x*_O_4_/SiO_2_@l-Arg, (d) Fe_3−*x*_Bi_*x*_O_4_/SiO_2_@l-ArgEt_3_^+^I^−^ and (e) Fe_3−*x*_Bi_*x*_O_4_/SiO_2_@l-ArgEt_3_^+^I^−^/Zn(ii).

The magnetic characteristics of the Fe_3−*x*_Bi_*x*_O_4_ core and each shell of the bionanostructure Fe_3−*x*_Bi_*x*_O_4_/SiO_2_@l-ArgEt_3_^+^I^−^/Zn(ii) were studied through VSM, as shown in Fig. 5. According to the data, the curves belong to (a) Fe_3−*x*_Bi_*x*_O_4_, (b) Fe_3−*x*_Bi_*x*_O_4_/SiO_2_, (c) Fe_3−*x*_Bi_*x*_O_4_/SiO_2_@l-Arg, (d) Fe_3−*x*_Bi_*x*_O_4_/SiO_2_@l-ArgEt_3_^+^I^−^, and (e) Fe_3−*x*_Bi_*x*_O_4_/SiO_2_@l-ArgEt_3_^+^I^−^/Zn(ii), exhibiting the saturation magnetization values of 18.508 emu g^−1^, 16.603 emu g^−1^, 7.701 emu g^−1^, 11.010 emu g^−1^, and 11.691 emu g^−1^, respectively. The results confirmed that not only the core but also the final bionanocomposite possessed magnetic properties. Embedding the silica and l-Arginine layers on the core decreased its magnetization due to the covering of the magnetized core with diamagnetic layers. Surprisingly, creating the l-ArgEt_3_^+^I^−^ ionic liquid increased the saturation magnetization value (curve d) in comparison to the former layer (curve c). This increase was also observed by embedding Zn(ii) on the outer layer of the bionanocomposite (curve e).

**Fig. 6:**
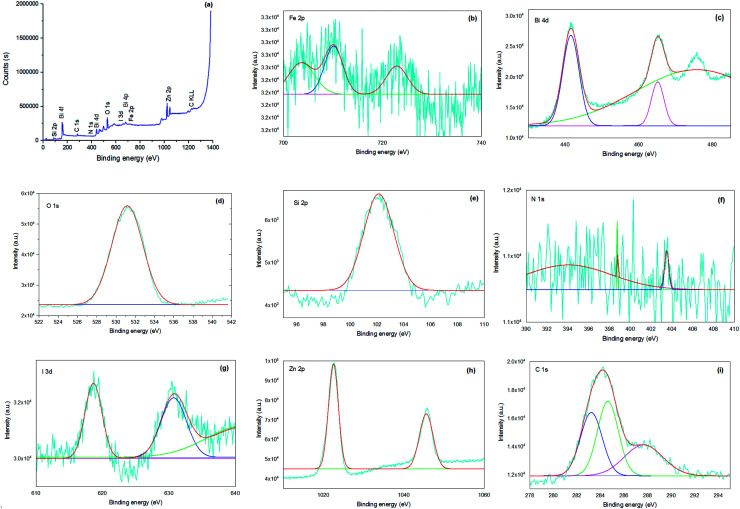
XPS survey spectra of Fe_3−*x*_Bi_*x*_O_4_-SiO_2_@l-ArgEt_3_^+^I^−^ Zn(ii) (a) and high-resolution XPS spectra of Fe 2p (b), Bi 4d (c), O 1s (d), Si 2p (e), N 1s (f), I 3d (g), Zn 2p (h), and C 1s (i).

X-ray photoelectron spectroscopy (XPS) was performed on the bionanocomposite to clarify its chemical composition and elemental valence states (Fig. 6). In the survey spectrum of the sample (Fig. 6a), the presence of Si, C, N, Bi, O, Fe, Zn, and I in Fe_3−*x*_Bi_*x*_O_4_/SiO_2_@l-ArgEt_3_^+^I^−^/Zn(ii) was confirmed through the appearance of peaks at the binding energies of 102 eV (Si 2p), 163 eV (Bi 4f), 287 eV (C 1s), 399 eV (N 1s), 439 eV (Bi 4d), 531 (O 1s), 628 (I 3d), 678 (Bi 4p), 726 (Fe 2p), 1022 (Zn 2p_3/2_), and 1045 (Zn 2p_1/2_). The atomic ratio of the elements of the bionanostructure are Zn (12.02%), Si (6.02%), Fe (49.22%), I (0.43%), and Bi (5.40%). The chemical states of Fe, Bi, O, Si, N, I, Zn, and C are displayed in their corresponding XPS spectra, as shown in Fig. 6b–i, respectively. According to Fig. 6b, the peak of Fe 2p_3/2_ at 710.15 eV revealed that both Fe^2+^ and Fe^3+^ are present in the bismuthmagnetite core. According to the previous reports, the binding energy of Fe 2p_3/2_ is about 709 eV for Fe^2+^ and 711 eV for Fe^3+^.^54^ The Fe 2p_1/2_ peak appeared at 722.89 eV. The Fe 2p_1/2_ and Fe 2p_3/2_ peaks of elemental iron [Fe(0)] should appear at 720 eV and 707 eV, respectively.^55^ Thus, there was no Fe(0) in the bionanocomposite. According to Fig. 6c, the relative symmetrical peaks at 441.58 eV (Bi 4d_5/2_) and 465.15 eV (Bi 4d_3/2_) with the splitting of 23.57 eV confirmed the presence of Bi(iii) in the structure.^56^ According to the peak at 531.17 eV in Fig. 6d, the O^2−^ species is present in the structure.^57^ The Si 2p peak in Fig. 6e at the binding energy of 102.129 eV confirmed the presence of SiO_2_ in the bionanocomposite. Fig. 6f presents the detailed XPS analysis of N 1s. According to the data, the peaks at 394.06 eV (C–N=), 398.74 eV (C–N), and 403.50 (C=N) verified the presence of the argininium core in the IL layer of the bionanocomposite.^58^ However, the peak in the region of 401.5–405 eV, which is related to the interactions of carbon and nitrogen, could be affected by another neighboring nitrogen.^59^ The absence of a peak at 399.4 eV, which corresponds to the NH_2_ group,^60^ can be due to the ammonium triethyl iodide moiety of the IL, and also the coordination of the nitrogen part of the ionic liquid with the Zn(ii) outer layer of the bionanocomposite. The two peaks at 618.63 eV (I 3d_5/2_) and 603.72 eV (I 3d_3/2_) in Fig. 6g validate the presence of I^+^ in the nanostructure.^61^ As shown in Fig. 6h, the peaks at 1022.48 eV (Zn 2p_3/2_) and 1045.57 eV (Zn 2p_1/2_) are attributed the presence of Zn^2+^ in the bionanocomposite. The characteristic peak of elemental Zn at 1021 eV was not observed.^62^ As shown in Fig. 6i, in the C 1s diagram, the peaks at 284.01 eV, 285.62 eV, and 287.67 eV correspond to the sp^2^ carbon, sp^3^ carbon, and the C–N and C=O moieties, respectively.^63^

**Fig. 7:**
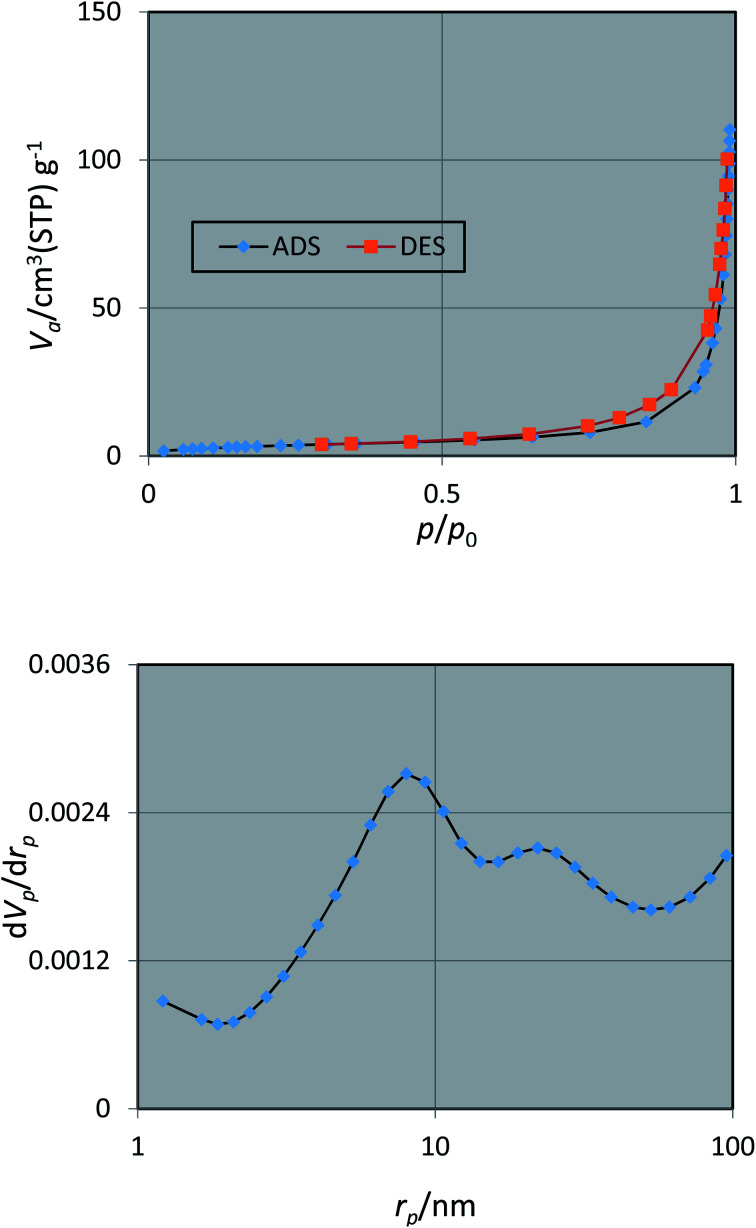
Nitrogen adsorption/desorption isotherms (top) and pore size distribution (down) of nano Fe_3−*x*_Bi_*x*_O_4_/SiO_2_@l-ArgEt_3_^+^I^−^/Zn(ii).

The porous nature of the prepared nano Fe_3−*x*_Bi_*x*_O_4_/SiO_2_@l-ArgEt_3_^+^I^−^/Zn(ii) was investigated *via* N_2_ adsorption/desorption isotherm (top) and pore size distribution (bottom) measurements, as shown in Fig. 7. According to the ADS/DES isotherm (top), the Fe_3−*x*_Bi_*x*_O_4_/SiO_2_@l-ArgEt_3_^+^I^−^/Zn(ii) nanostructure can be classified as a type III isotherm (according to IUPAC classification), which illustrated a type H3 hysteresis loop in the *p*/*p*_0_ range of 0.55–0.97, corresponding to slit-shaped pores. The physicochemical properties of the nanostructure are as follows: BET surface area (*a*_s,BET_) = 12.687 m^2^ g^−1^, total pore volume = 0.1699 cm^3^ g^−1^, and mean pore diameter = 53.58 nm. The data from the BJH pot (down) are *r*_p,peak_ (area) = 7.99 nm, *a*_p_ = 15.43 m^2^ g^−1^, and *v*_p_ = 0.1705 cm^3^ g^−1^.

### Investigation of the catalytic activity of the nano Fe_3−*x*_Bi_*x*_O_4_/SiO_2_@l-ArgEt_3_^+^I^−^/Zn(ii)

To optimize the reaction conditions, the five-component one-pot reaction of 1,2-phenylenediamine (1a, 1 mmol), Meldrum's acid (2, 1.2 mmol), malononitrile (3, 1.2 mmol), 4-nitrobenzaldehyde (4c, 1.2 mmol) and cyclohexyl isocyanide (5a, 1.2 mmol) was chosen as a model reaction. According to the data in Table 2, different parameters were checked to obtain 2-(2-(cyclohexylamino)-2-(1,2,4,5-tetrahydro-2,4-dioxobenzo[*b*][1,4]diazepin-3-ylidene)-1-(4-nitrophenyl)ethyl)malononitrile (6d). The reaction was examined under solvent-free conditions (entries 1 and 2) and also utilizing H_2_O and EtOH (entries 3 and 4, respectively). The results showed that EtOH seems to be a good choice. Performing the reaction at 60 °C did not produce good results (entry 2). The catalyst amount was another investigated parameter (entries 5–8). The presence of 0.012 g of nano Fe_3−*x*_Bi_*x*_O_4_/SiO_2_@l-Arg-Et_3_^+^I^−^/Zn(ii) was sufficient (entry 8). To detect the effect of the mixed green solvent of EtOH–H_2_O (1 : 1), it was examined at room temperature and at 50 °C (entries 9 and 10, respectively). Polyethylene glycol was another solvent choice that did not produce a good result (entry 11). To check the media temperature, the preparation of 6d was examined in an ice-bath, which did not yield any product (entry 12). It must be mentioned that in each case, the reaction was carried out for up to 24 h, but no further improvement occurred after the reported durations, as shown in Table 2.

**Table 2:** Screening the reaction parameters for the synthesis of 6d

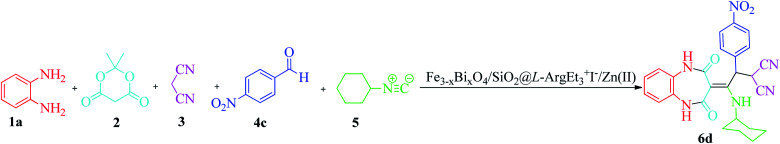			
Entry	Fe_3−*x*_Bi_*x*_O_4_/SiO_2_@l-ArgEt_3_^+^I^−^/Zn(ii) (g)/solvent (3 mL)/temperature (°C)	Time (h)	Yield (%)
1	0.032/—/rt	24	32
2	0.032/—/60	12	20
3	0.032/H_2_O/rt	16	10
4	0.032/EtOH/rt	16	62
5	0.04/EtOH/rt	12	51
6	0.02/EtOH/rt	16	67
7	0.024/EtOH/rt	20	67
8	0.012/EtOH/rt	9	83
9	0.012/EtOH–H_2_O (1 : 1)/rt	12	10
10	0.012/EtOH–H_2_O (1 : 1)/50	10	40
11	0.012/PEG (2000)/rt	10	5
12	0.012/EtOH/0	20	—

To evaluate the effect of the presence of the bionanocomposite on promoting the model reaction, the preparation of the product 6d was examined in the absence of the catalyst, and also each layer of Fe_3−*x*_Bi_*x*_O_4_/SiO_2_@l-ArgEt_3_^+^I^−^/Zn(ii) under the optimized reaction conditions for 9 h. The data is presented in Table 3. The model reaction was also examined in the presence of the l-ArgEt_3_^+^I^−^ ionic liquid (entry 7) and only EtI (entry 8). According to the data, in the case of using the ionic liquid, many by-products were observed during the reaction (9 h). The observations confirmed that the presence of each shell on the bismuthmagnetite core affected the reaction progress. This can be ascribed to the combined catalytic effect of each component in the whole bionanocomposite, which synergically extended its catalytic potential.

**Table 3:** Investigation of the catalytic role of Fe_3−*x*_Bi_*x*_O_4_-SiO_2_@l-ArgEt_3_^+^I^−^/Zn(ii) in the synthesis of 6d

Entry	Catalyst (0.012 g)/EtOH (3 mL)/rt	Yield (%)
1	—	∼5
2	Fe_3−*x*_Bi_*x*_O_4_	12
3	Fe_3−*x*_Bi_*x*_O_4_/SiO_2_	23
4	Fe_3−*x*_Bi_*x*_O_4_/SiO_2_@l-Arg	30
5	Fe_3−*x*_Bi_*x*_O_4_/SiO_2_@l-ArgEt_3_^+^I^−^	42
6	Fe_3−*x*_Bi_*x*_O_4_/SiO_2_@l-ArgEt_3_^+^I^−^/Zn(ii)	83
7	l-ArgEt_3_^+^I^−^	10
8	EtI	—

Based on the optimized reaction conditions, different derivatives of the 1,2,4,5-tetrahydro-2,4-dioxobenzo[*b*][1,4]diazepine malononitrile were successfully prepared according to the data in Table 4 (method A). 1,2-Phenylenediamine 1a reacted with benzaldehyde and its derivatives (4a–d) in the presence of cyclohexyl isocyanide 5a to obtain their corresponding products within 9–17 h in 66–91% yield (entries 1, 2, 4, and 5, respectively). *t*-Butyl isocyanide 5b achieved satisfactory results as another alkyl isocyanide candidate (entry 3). Terephthalaldehyde, as a bifunctional aldehyde resulted in the formation of 2-(2-(cyclohexylamino)-2-(2,4-dioxo-4,5-dihydro-1*H*-benzo[*b*][1,4]diazepin-3(2*H*)-ylidene)-1-(4-formylphenyl)ethyl)malononitrile 6f chemoselectively, as only one of the aldehydic moieties reacted (entry 6). The reaction was performed in the presence of 1a, 2, 3, 4i, and 5a (in a 1 : 1.2 : 1.2 : 0.6 : 1.2 molar ratio) and nano Fe_3−*x*_Bi_*x*_O_4_/SiO_2_@l-ArgEt_3_^+^I^−^/Zn(ii) (0.012 g). Performing the reaction in the presence of 4-methyl-1,2-phenylenediamine 1b yielded 6g successfully. To further improve the results, the reaction was examined under sonication and the results are summarized in Table 3 (method B). As seen, the reaction times decreased significantly (20–60 min), but an overall increase in the yield was not observed, which can be explained by the cavitation effects of ultrasound. The known compounds were characterized by comparing the obtained data with their authentic reports.^64^ The spectral data of the new derivatives (6e and 6f) are presented in the Experimental section.

**Table 4:** Synthesis of 1,2,4,5-tetrahydro-2,4-dioxobenzo[*b*][1,4]diazepine malononitriles 6a–g catalyzed by nano Fe_3−*x*_Bi_*x*_O_4_/SiO_2_@l-ArgEt_3_^+^I^−^/Zn(ii) (0.012 g) in EtOH (3 mL)

Entry	Product	Method A	Method B
		Time (h)/yield (%)	Time (min)/yield (%)
1	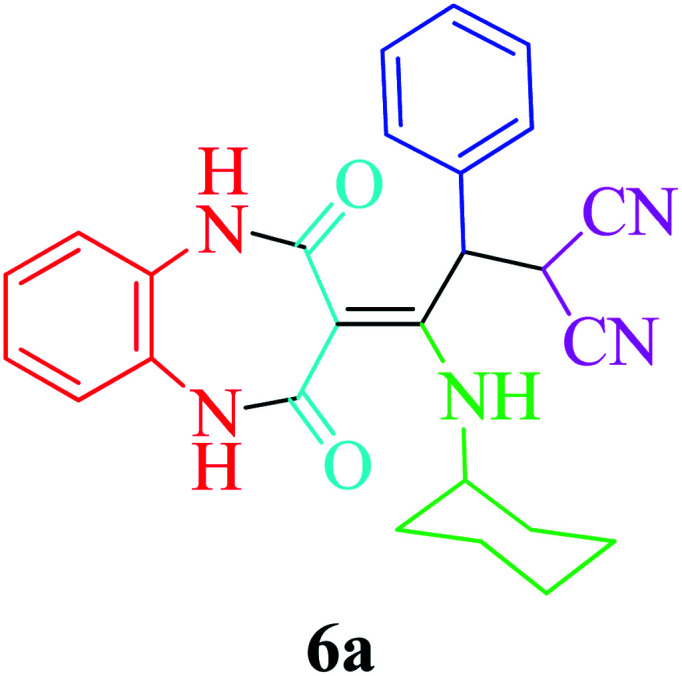	13/91	50/62
2	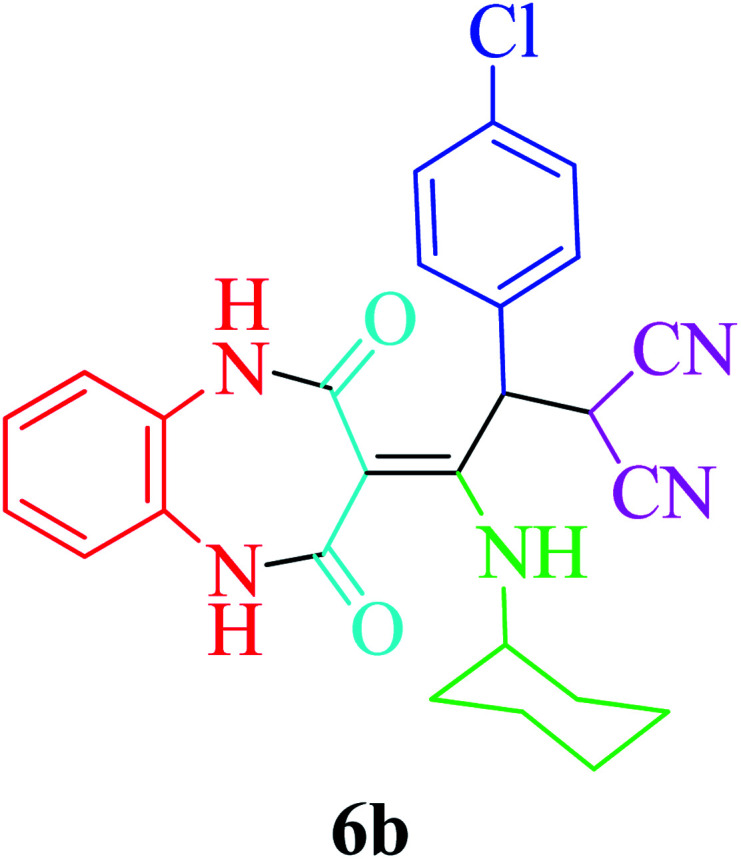	15/66	50/72
3	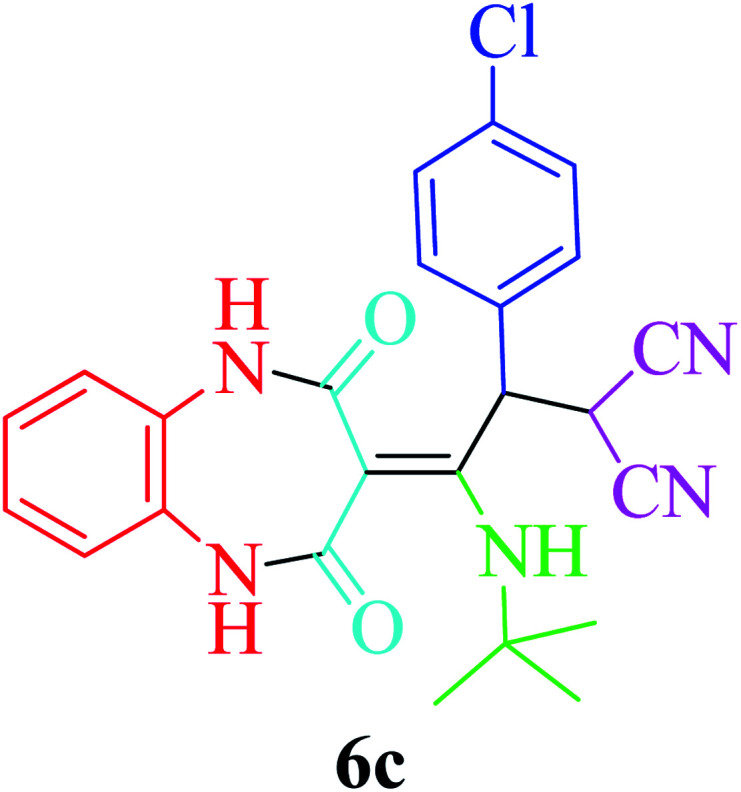	15/72	50/74
4	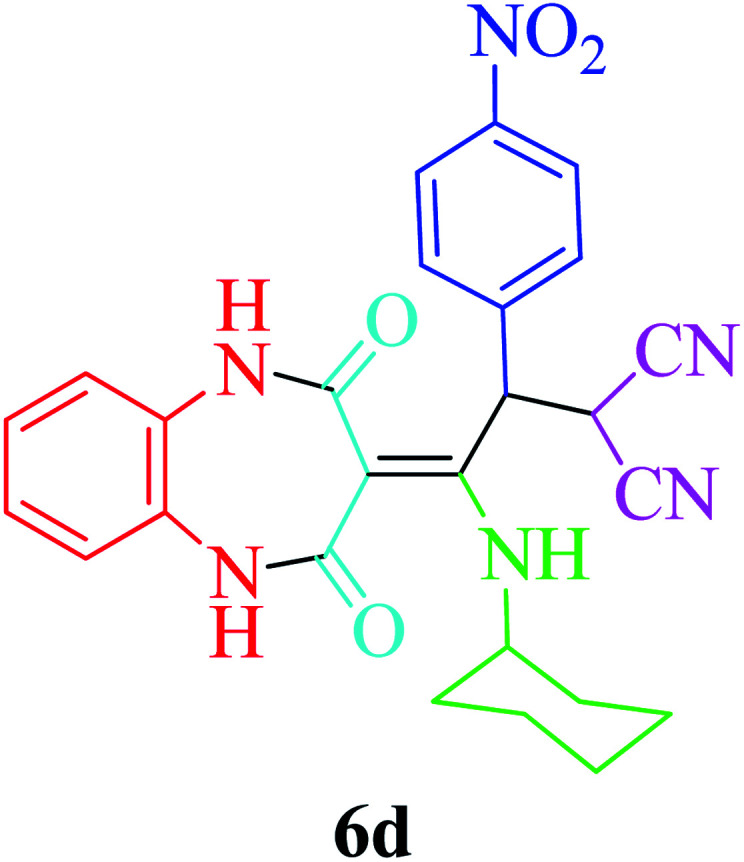	9/83	60/71
5	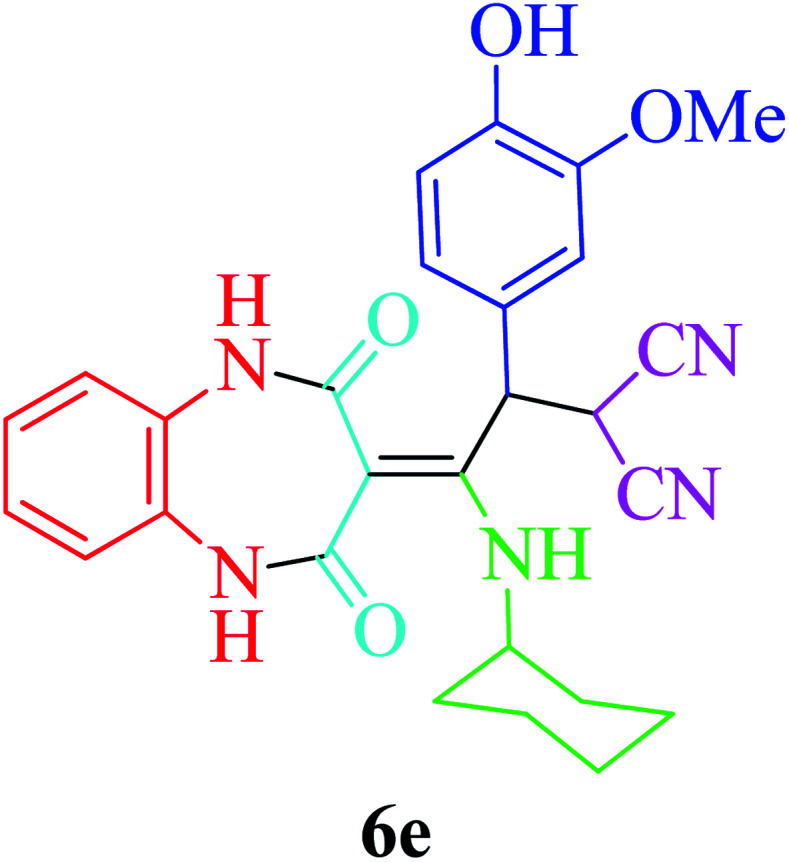	17/73	50/84
6	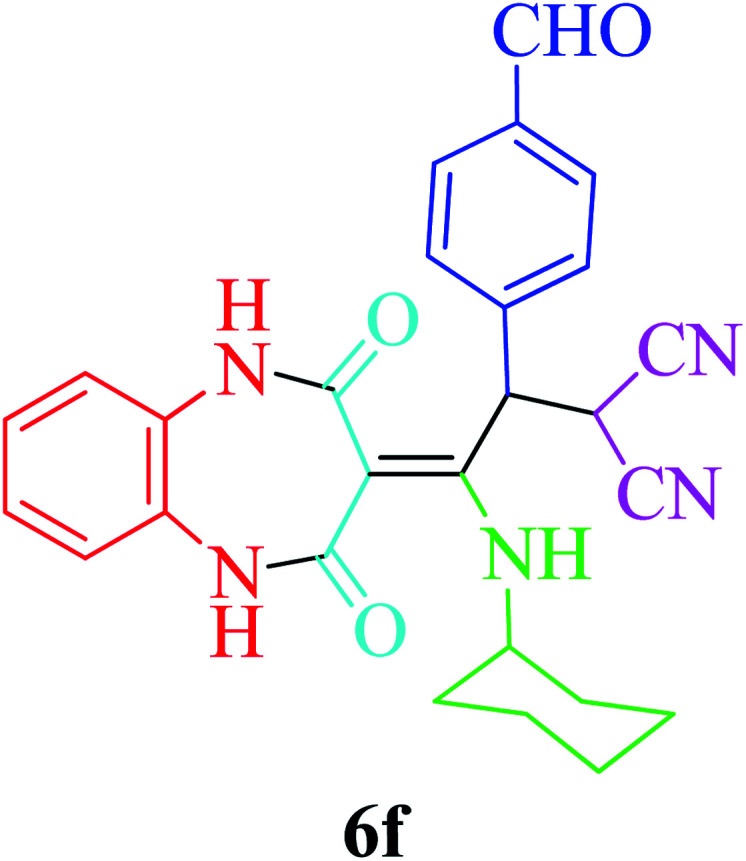	17/77	20/63
7	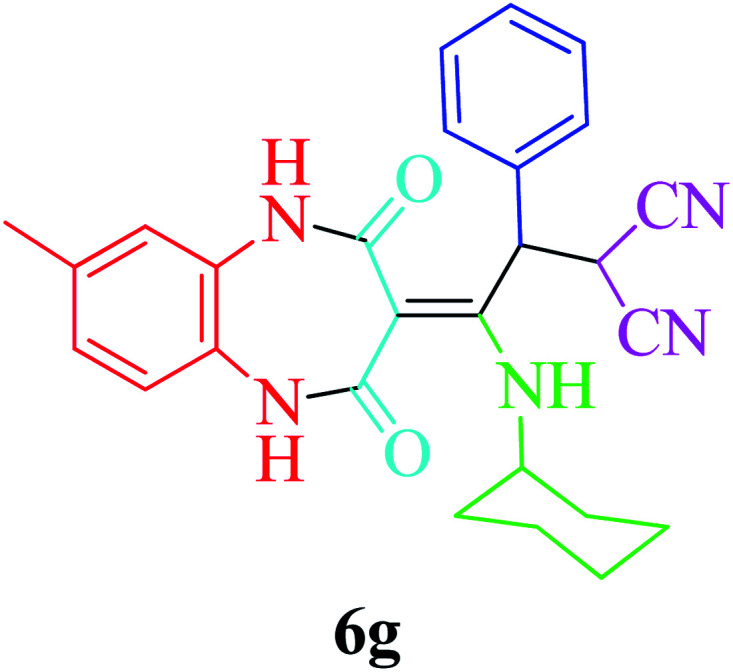	12/96	20/73

Furthermore, to extend the efficacy of the novel nano Fe_3−*x*_Bi_*x*_O_4_/SiO_2_@l-ArgEt_3_^+^I^−^/Zn(ii) the preparation of malonamides 7a–f through a pseudo six-component condensation was performed successfully under the optimal reaction conditions within 10–19 h in 65–98% yield Table 5 (method A). Heterocycles 7a–e were obtained through the reaction of 1,4-phenylenediamine 1c, Meldrum's acid 2, malononitrile 3, aldehydes 4f–h, and isocyanides 5a and b (entries 1–5). Indole-3-carbaldehyde 4i, as a fused bicyclic heteroaromatic aldehyde candidate, achieved the corresponding adduct 7f within 17 h (entry 6). Concerning the results, performing the reactions under sonication decreased the duration notably (Table 5, method B). Also, no significant substituent effect was demonstrated. The known compounds were characterized by comparing their data with the literature.^64^ The spectral data of product 7f is displayed in the Experimental section.

**Table 5:** Synthesis of malonamides 7a–f in the presence of nano Fe_3−*x*_Bi_*x*_O_4_/SiO_2_@l-ArgEt_3_^+^I^−^/Zn(ii) (0.012 g) in EtOH (3 mL)

Entry	Product	Method A	Method B
		Time (h)/yield (%)	Time (min)/yield (%)
1	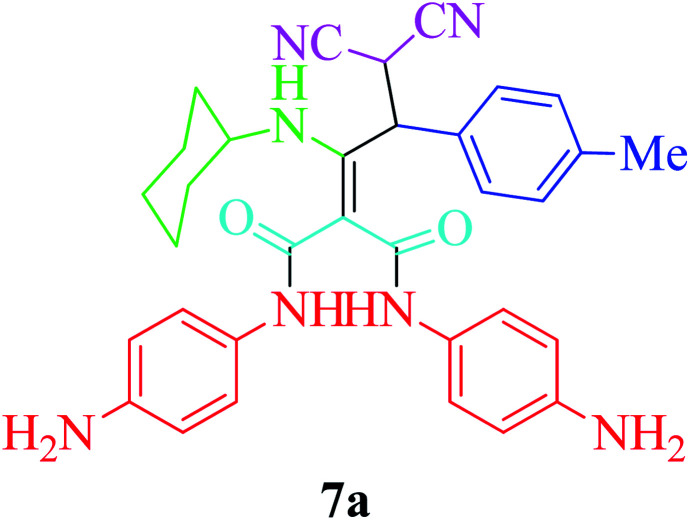	19/72	50/87
2	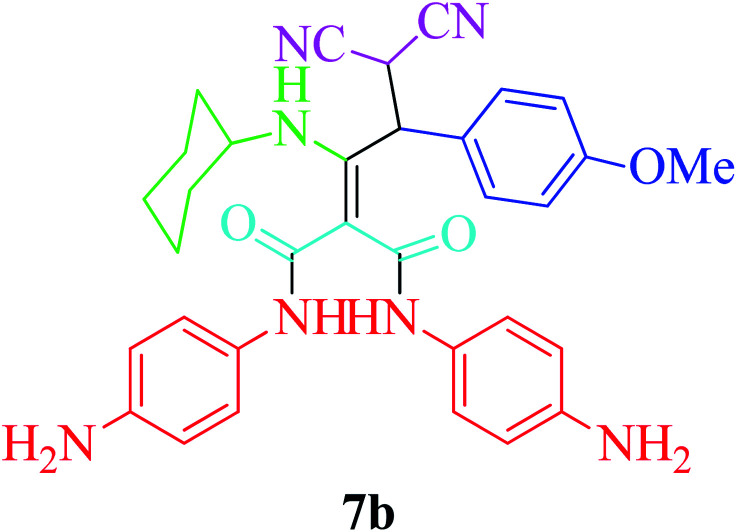	10/98	50/82
3	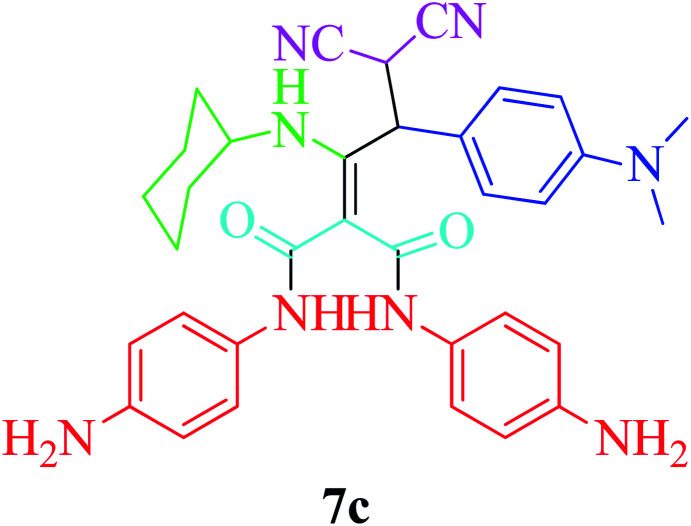	10/91	20/90
4	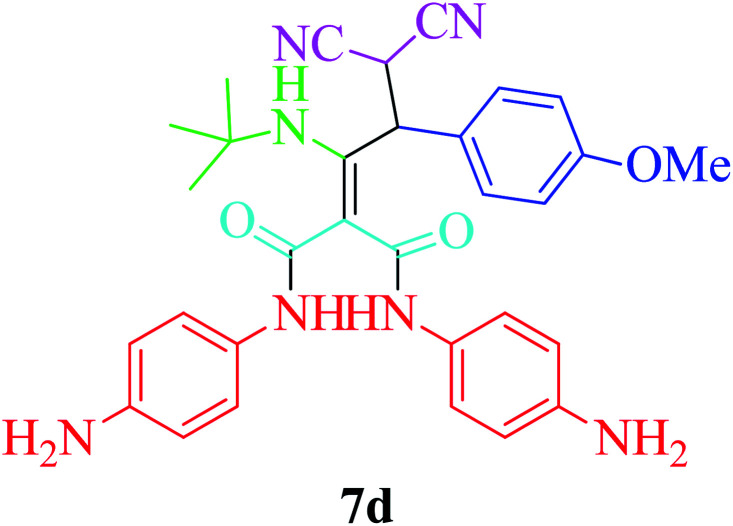	10/98	40/70
5	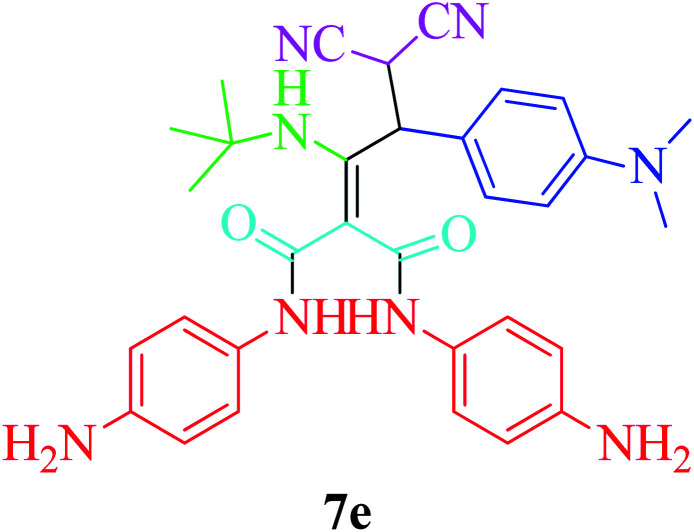	12/72	50/71
6	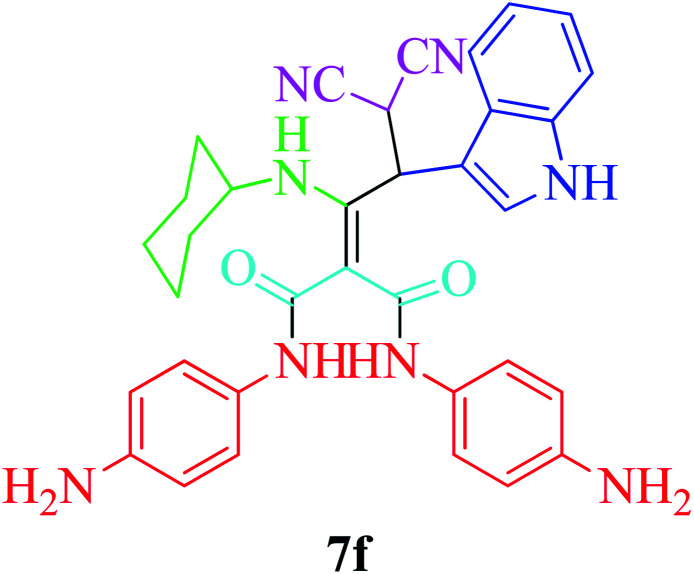	17/65	60/62

**Fig. 8:**
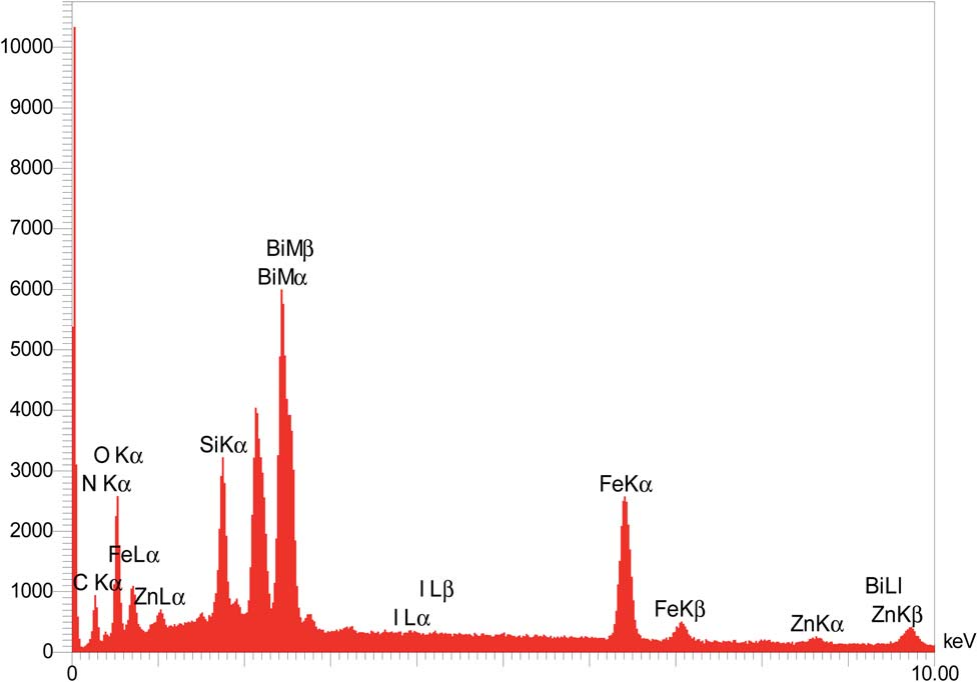
EDAX analysis of the reused Fe_3−*x*_Bi_*x*_O_4_/SiO_2_@l-ArgEt_3_^+^I^−^/Zn(ii).

**Fig. 9:**
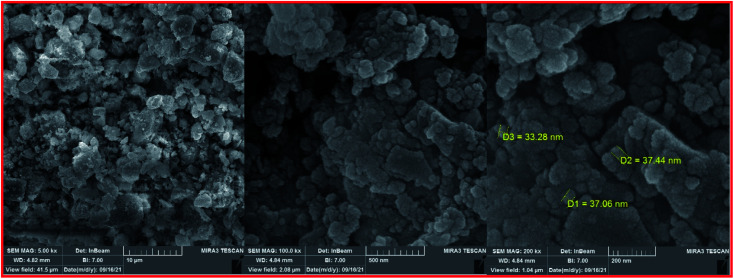
FESEM images of the recovered Fe_3−*x*_Bi_*x*_O_4_/SiO_2_@l-ArgEt_3_^+^I^−^/Zn(ii).

To clarify the possibility of recycling and reusing the bionanostructure, the model reaction was examined with 2 runs. Product 4d was obtained in 82% and 80% yield in the first and second runs, respectively. After each cycle, the bio nano core–shell was separated with an external magnet, washed with MeOH (2 × 3 mL), and air-dried. The reused catalyst was characterized thought EDAX analysis (Fig. 8) and FESEM imaging (Fig. 9). The EDAX data confirmed that Fe_3−*x*_Bi_*x*_O_4_/SiO_2_@l-ArgEt_3_^+^I^−^/Zn(ii) consisted of iron (17.91 wt%), bismuth (30.45 wt%), oxygen (23.09 wt%), silicon (16.25 wt%), carbon (9.05 wt%), nitrogen (3.16 wt%), iodine (0.08 wt%), and zinc (0.13 wt%). The FESEM spectra also demonstrated that some agglomeration happed in the bionanocomposite compared to the fresh nanocatalyst. The average size of the nanoparticles reached 30–40 nm.

**Scheme 3:**
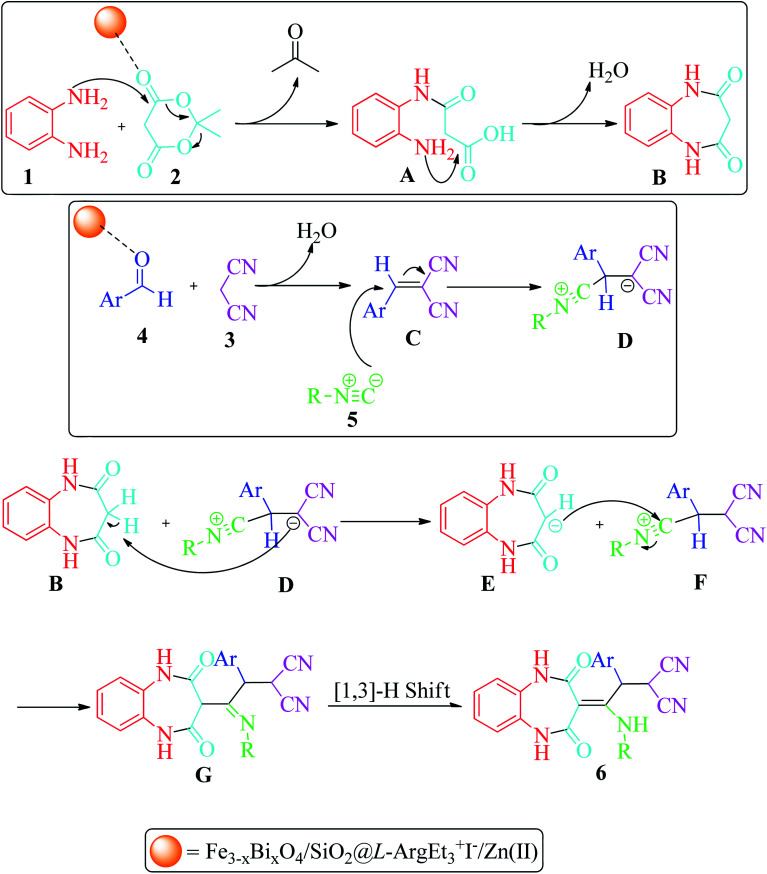
Proposed mechanism for the formation of 1,2,4,5-tetrahydro-2,4-dioxobenzo[*b*][1,4]diazepines.

The probable mechanism for the synthesis of 1,2,4,5-tetrahydro-2,4-dioxobenzo[*b*][1,4]diazepine derivatives 6 is shown in Scheme 3. The condensation reaction of 1,2-phenylenediamines 1 with Meldrum's acid 2 in the presence of nano Fe_3−*x*_Bi_*x*_O_4_-SiO_2_@l-ArgEt_3_^+^I^−^/Zn(ii) through release of acetone and aqua formed the intermediates 3-((2-aminophenyl)amino)-3-oxopropanoic acid A and benzodiazepine-2,4-dione B, respectively. Alternatively, 2-(arylomethylene)malononitrile C was formed *via* nucleophilic attack of malononitrile 3 to activated aldehyde 4 through a condensation reaction. The nucleophilic attack of isocyanide 5 to C*via* a Michel-type addition reaction led to intermediate D, which absorbed a proton from the acidic methylene of B to form dioxo-tetrahydro-1*H*-benzodiazepin-3-ides E and F, respectively. The nucleophilic attack of E to F yielded G, which followed by [1,3]-H shift (analogues to imine-enamine tautomerization), produced the final products 6.

In the final part, the comparison of the data for the synthesis of 2-(2-(cyclohexylamino)-2-(2,4-dioxo-4,5-dihydro-1*H*-benzo[*b*][1,4]diazepin-3(2*H*)-ylidene)-1-(4-nitrophenyl)ethyl)malononitrile 6d is provided in Table 6. The significant feature of the current report is the remarkable reduction in the reaction time, not only with the traditional stirring method but also under sonication (comparing entries 2 and 3 with 1). The yield enhancement is another noteworthy aspect of the present procedure. Performing the mentioned MCR in green media (EtOH) is another important feature of this report compared to the previous route (which utilized CH_2_Cl_2_ as the solvent).

**Table 6:** Comparison of the synthesis of 6d with the previously reported procedure

Entry	Conditions	Time/yield (%)	Ref.
1	CH_2_Cl_2_/rt	24 h/48	60
2	Fe_3−*x*_Bi_*x*_O_4_/SiO_2_@l-ArgEt_3_^+^I^−^/Zn(ii) (0.012 g)/EtOH (3 mL)/rt	9 h/83	This work
3	Fe_3−*x*_Bi_*x*_O_4_/SiO_2_@l-ArgEt_3_^+^I^−^/Zn(ii) (0.012 g)/EtOH (3 mL)/rt/Us	60 min/71	This work

## Experimental

### General

The utilized chemicals were obtained from Aldrich, Merck, and Alfa-Aesar and used as received without further purification. Melting points were obtained using an Electrothermal 9200 apparatus and reported uncorrected. FT-IR spectra were obtained using KBr disks of the samples on a Bruker FT-IR (Tensor 27) spectrometer. Magnetization properties were measured using a VSM (LBKFB model) apparatus. Homogenization of the nanostructure was performed in a WiseClean (power of 90 W). Morphology and size were estimated *via* FESEM (VEGA\\TESCAN-LMU). Mass spectra were obtained using a Gc-Mass 5973 network mass-selective detector on a Gc6690 Agilent device. The EDAX analysis was performed using a TESCAN MIRA III machine. Elemental investigations were performed using a Philips-PW2404 XRF spectrometer. A UNIVERSAL 320 centrifuge apparatus (5000–10 000 rpm) was utilized for the preparation of the nanostructure. The XPS analysis was performed using a PHOIBOS machine with a Zr X-ray source (1486.71 eV). The peak fitting was performed using SpecsLab Prodigy Version 4.43.2-r73078. BET measurements were performed on a Belsorp Mini Japan, Finetech 40 point apparatus. The ICP-OES analysis was done using a Spectro Arcos instrument. Sonication was performed using an HD 3100 ultrasonic homogenizer (Bandelin Company, Germany). A standard horn SH 70 G emitting 20 kHz ± 500 Hz ultrasound at intensity levels tunable up to a maximum sonic power density of 100 W cm^−2^ was used. Sonication was carried out at 36% amplitude. An MS73 probe of 13 mm diameter was immersed directly into the reaction mixture.

### Synthetic procedure for Fe_3−*x*_Bi_*x*_O_4_/SiO_2_@l-ArgEt_3_^+^I^−^/Zn(ii) bionanocomposite

#### Synthesis of nano bismuthmagnetite (Fe_3−*x*_Bi_*x*_O_4_)

The bismuthmagnetite nanostructure was prepared through a modified precipitation–oxidation method, which was reported previously for the synthesis of Fe_3−*x*_Cr_*x*_O_4_.^65^ In a typical procedure, solutions of FeSO_4_·7H_2_O (0.9 M) and BiCl_3_ (0.9 M) were prepared in HCl solution (1 M). In a flask, to 10 mL of the prepared solution of Fe^2+^ and Bi^3+^ in equal amounts, hydrazine hydrate (1 mL) was added to reach pH < 1 to prevent the oxidation of Fe^2+^ and hydroxide precipitation. The resulting solution was refluxed for 30 min at 90 °C under an N_2_ atmosphere (to prevent oxidation of iron by air). In the next step, a mixture of NaOH (4 M, 5 mL) and NaNO_3_ (0.9 M, 5 mL) was added dropwise (within 5 min) to the reaction mixture and stirred magnetically for 2 h at 90 °C. The mixture was cooled, the residue separated through an external magnet and washed with water (3 × 10 mL). Air-drying the solid for 10 h yielded Fe_3−*x*_Bi_*x*_O_4_ as a black solid.

#### Preparation of nano Fe_3−*x*_Bi_*x*_O_4_/SiO_2_

The process was accomplished through a modified procedure.^51*e*^ Typically, a mixture of Fe_3−*x*_Bi_*x*_O_4_ nanoparticles (1 g), was dissolved in a mixture of 25 wt% ammonia (2 mL), deionized water (20 mL) and absolute ethanol (60 mL) and sonicated in a sonic-bath for 30 min. Then a solution TEOS (0.5 mL) in absolute ethanol (1 mL) was added dropwise to the above mixture and stirred continuously for 20 h at ambient temperature. Finally, the was solid separated using an external magnet and washed with absolute ethanol (3 × 5 mL). The product was dried at 70 °C for 5 h to produce nano Fe_3−*x*_Bi_*x*_O_4_/SiO_2_.

#### Embedding l-Arginine amino acid on nano Fe_3−*x*_Bi_*x*_O_4_/SiO_2_ layer (Fe_3−*x*_Bi_*x*_O_4_/SiO_2_@l-Arg)

To a mixture of nano Fe_3−*x*_Bi_*x*_O_4_/SiO_2_ (1 g) and l-arginine (1 g) in acetonitrile (10 mL), diluted H_2_SO_4_ (0.01 M) was added dropwise to regulate the pH to ∼4. The mixture was refluxed for 7 h. The residue was separated using an external magnet and washed with a mixture of acetonitrile/absolute ethanol (1 : 1) (2 × 10 mL). The obtained solid was dried at 100 °C for 4 h to get Fe_3−*x*_Bi_*x*_O_4_/SiO_2_@l-Arg.

#### *In situ* fabrication of triethylargininium iodide on the silicated-bismuthmagnetite core (Fe_3−*x*_Bi_*x*_O_4_/SiO_2_@l-ArgEt_3_^+^I^−^)

A mixture of Fe_3−*x*_Bi_*x*_O_4_/SiO_2_@l-Arg nanocomposite (1 g) and ethyl iodide (2 g) in a water/ethanol (1 : 1) mixture (20 mL) was refluxed at 90 °C for 3 h. After completion and cooling, the residue was separated using an external magnet and washed with water/absolute ethanol (1 : 1) (3 × 10 mL). The black solid was dried at 50 °C for 2 h to obtain Fe_3−*x*_Bi_*x*_O_4_/SiO_2_@l-ArgEt_3_^+^I^−^.

#### Preparation of nano Fe_3−*x*_Bi_*x*_O_4_/SiO_2_@l-ArgEt_3_^+^I^−^/Zn(ii)

A mixture of Fe_3−*x*_Bi_*x*_O_4_/SiO_2_@l-ArgEt_3_^+^I^−^ nanostructure (1 g) and zinc acetate (1 g) in absolute ethanol (20 mL) was refluxed for 7 h. Next, the solid was separated using an external magnet and washed with absolute ethanol (3 × 10 mL). After air-drying the solid residue for 2 h followed by heating in an oven 50 °C for 2 h, the final bionanocomposite Fe_3−*x*_Bi_*x*_O_4_/SiO_2_@l-ArgEt_3_^+^I^−^/Zn(ii) was obtained.

#### Preparation of l-ArgEt_3_^+^I^−^ ionic liquid

The ionic liquid was prepared based on a previously reported procedure with some modifications.^33*c*^ To a mixture of l-arginine (1.74 g, 10 mmol) in a water/absolute ethanol mixture (1 : 1) (6 mL), EtI (4.679 g, 30 mmol) was added and refluxed for 7 h. Then, the mixture was cooled to room temperature. The mixture was concentrated through mild heating, followed by the addition of MeOH (5 mL), vaporizing, and air-drying. The pure l-ArgEt_3_^+^I^−^ ionic liquid was obtained as a pale blue viscous liquid. IR (KBr, cm^−1^): 3336, 3161, 2968, 1647, 1454, 1396, 1043, 871, 790, 663, 549. ^1^H NMR (300 MHz, DMSO-*d*_6_): 0.98 (brs, 9H, 3CH_3_), 1.11 (m, 2H, CH_2_), 1.52 (m, 2H, CH_2_), 2.49 (brs, 1H, NH), 2.50–3.09 (m, 4H, CH_2_), 3.36–3.9 (m, 5H, 2CH_2_ + CH), 7.44 (brs, 4H, NH + OH). ^13^C NMR (75 MHz, DMSO-*d*_6_): 11.33, 14.00, 18.63, 24.83, 26.55, 41.15, 45.05, 48.74, 56.17, 157.12, 171.48. MS (ESI) *m*/*z* 386 [M]^+^, 357 ([M]^+^–Et), 343 ([M]^+^–Me, –Et), 327 ([M]^+^–2Et), 283 ([M]^+^–3Et, –CO_2_H), 267 ([M]^+^–2Et, –Me, –CO_2_H), 253 ([M]^+^–3Et, –CO_2_H), 175 [Arg]^+^, 156 [Arg–NH_2_]^+^, 113 [Arg–NH_2_, –CO_2_]^+^, 100 [NHEt_3_]^+^, 84 [CH_2_CH_2_NHCNHNH_2_]^+^, 56 [guanidinium]^+^, 44 [CO_2_].

### General procedure for the synthesis of 1,2,4,5-tetrahydro-2,4-dioxobenzo[*b*][1,4]diazepine malononitriles 6a–g*via* five-component reaction

To a solution of aromatic 1,2-diamines (1a–b, 1 mmol), Meldrum's acid (2, 1.2 mmol), malononitrile (3, 1.2 mmol), aromatic aldehydes (4a–e, 1.2 mmol), and isocyanides (5a–b, 1.2 mmol) in ethanol (3 mL), the nano Fe_3−*x*_Bi_*x*_O_4_/SiO_2_@l-ArgEt_3_^+^I^−^/Zn(ii) (0.012 g) was added. This mixture was stirred magnetically at room temperature (method A) or under sonication (100 W cm^−2^) (method B) for the appropriate time and monitored by TLC (*n*-hexane/EtOAc 1 : 1). After completion, the bionanocatalyst was separated using an external magnet. The pure products 6a–g were obtained by preparative thin-layer chromatography (PLC).

#### 2-(2-(Cyclohexylamino)-2-(2,4-dioxo-4,5-dihydro-1*H*-benzo[*b*][1,4]diazepin-3(2*H*)-ylidene)-1-(4-hydroxy-3-methoxyphenyl)ethyl)malononitrile (6e)

Brown powder; m. p. = 80 °C; IR (KBr): 3445, 2926, 2854, 2194, 1736, 1700, 1650, 1559, 1521, 1457, 1396, 1339, 1034, 818, 748 cm^−1^. ^1^H NMR (300 MHz, DMSO-*d*_6_) *δ*: 0.9–1.22 (m, 6H, 3CH_2_), 1.39–1.63 (m, 4H, 2CH_2_), 1.92–2.00 (m, 1H, CH), 2.89 (brs, 1H, NH), 3.32 (s, 3H, OMe), 3.67–3.71 (m, 1H, CH), 3.92–3.97 (m, 1H, CH), 6.52–6.76 (m, 2H, Ar), 6.80–7.13 (m, 5H, Ar), 7.41 (brs, 1H, NH), 7.52–7.73 (m, 1H, OH), 7.99 (brs, 1H, NH). MS (ESI) *m*/*z* 485 [M]^+^, 355 ([M]^+^–cyclohexyl, –NH_2_, –OMe), 327 ([M]^+^–(methoxyphenyl)propanenitrilium), 281 ([M]^+^–hydroxy-methoxybenzylmalononitrile), 266 ([M]^+^–cyclohexyl, –NH_2_, –methoxyphenol), 237 ([M]^+^–cyclohexyl, –NH_2_, –methoxyphenol, –CN), 210 ([M]^+^–cyclohexyl, –NH_2_, –methoxyphenol, –2CN), 182 [methylene-1*H*-benzo[*b*][1,4]diazepine-2,4(3*H*,5*H*)-dione]^+^, 141 [methoxymethylphenol]^+^, 115 [*N*-methylcyclohexanamine]^+^, 98 [cyclohexanamine]^+^, 67 [malononitrile]^+^.

#### 2-(2-(Cyclohexylamino)-2-(2,4-dioxo-4,5-dihydro-1*H*-benzo[*b*][1,4]diazepin-3(2*H*)-ylidene)-1-(4-formylphenyl)ethyl)malononitrile (6f)

Cream powder; m. p. = 130 °C; IR (KBr): 3423, 2929, 2856, 1638, 1454, 1370, 1316, 1103, 898, 799, 750, 580, 468 cm^−1^;. ^1^H NMR (300 MHz, DMSO-*d*_6_) *δ*: 0.9–1.34 (m, 6H, 3CH_2_), 1.37–1.63 (m, 4H, 2CH_2_), 1.92–2.07 (m, 1H, CH), 2.82 (brs, 1H, NH), 3.16–3.67 (m, 2H, CH), 6.99–7.18 (m, 4H, Ar), 7.31–7.45 (m, 1H, Ar), 7.52–7.55 (m, 3H, Ar), 8.00 (brs, 1H, NH), 8.16 (brs, 1H, NH), 8.18 (s, 1H, aldehydic). MS (ESI) *m*/*z* 467 [M]^+^, 428 ([M]^+^–CH_2_CN), 405 ([M]^+^–CHCN_2_), 377 ([M]^+^–aniline), 357 ([M]^+^–phenylenediamine), 331 ([M]^+^–2CN, –cyclohexyl), 316 ([M]^+^–cyclohexylamine, –2CN), 206 [ethyl-1*H*-benzo[*b*][1,4]diazepine-2,4(3*H*,5*H*)-dione]^+^, 189 [methyl-1*H*-benzo[*b*][1,4]diazepine-2,4(3*H*,5*H*)-dione]^+^, 133 [ethylbenzaldehyde]^+^, 92 [toluene]^+^.

### General procedure for the synthesis malonamides 7a–f*via* pseudo six-component reaction

To a solution of para phenylenediamine (1c, 2 mmol), Meldrum's acid (2, 1 mmol), malononitrile (3, 1.1 mmol), aromatic aldehydes (4f–i, 1.1 mmol), isocyanides (5a–b, 1 mmol) in ethanol (3 mL), nano Fe_3−*x*_Bi_*x*_O_4_/SiO_2_@l-ArgEt_3_^+^I^−^/Zn(ii) (0.012 g) was added and the was mixture stirred at room temperature (method A) or under ultrasonication (100 W cm^−2^) (method B). After completion of the reaction, as observed by TLC (*n*-hexane/EtOAc 1 : 1), the catalyst was separated using an external magnet. The pure products 7a–f were obtained *via* the PLC technique.

#### *N* ^1^,*N*^3^-Bis(4-aminophenyl)-2-(3,3-dicyano-1-(cyclohexylamino)-2-(1*H*-indol-3-yl)propylidene)malonamide (7f)

Brown powder; m. p. = 145 °C; IR (KBr): 3567, 3421, 2925, 2854, 2169, 1650, 1542, 1514, 1421, 1069, 830, 745, 592, 516, 471, 520 cm^−1^. ^1^H NMR (300 MHz, DMSO-*d*_6_) *δ*: 0.84–1.9 (m, 10H, 5CH_2_), 3.74–3.83 (m, 3H, CH), 4.78–4.79 (brs, 4H, 2NH_2_), 6.13–6.55 (m, 7H, Ar), 6.56–6.71 (m, 3H, Ar), 6.90–6.99 (m, 3H, Ar), 7.47 (s, 1H, NH), 7.80–7.94 (m, 1H, NH), 10.84 (brs, 1H, NH), 10.87 (brs, 1H, NH). MS (ESI) *m*/*z* 586 [M]^+^, 405 ([M]^+^–CHCN_2_, –indolyl), 346 ([M]^+^–CO, –2phenylediamine), 331 ([M]^+^–phenylenediamine, –CO, –indolyl), 254 ([M]^+^–phenylenediamine, –CO, –indolyl, –cyclohexyl), 127 [*N*-ethylcyclohexanamine]^+^, 117 [indolyl]^+^, 108 [phenylenediamine]^+^, 99[cyclohexylamine]^+^, 91[aniline]^+^.

## Conclusions

In summary, a novel magnetic inorganic–bioorganic nanocomposite, Fe_3−*x*_Bi_*x*_O_4_/SiO_2_@l-ArgEt_3_^+^I^−^/Zn(ii), was successfully obtained *via* a multi-step simple synthetic procedure, which was characterized by the FT-IR, XRF, VSM, FESEM, EDAX, TGA/DSC, XPS, BET, and ICP-OES techniques. The nanoscale biocomposite was made up of a bismuthmagnetite core, which was covered by silica, triethylargininium iodide IL, and Zn(ii), respectively. The catalytic performance of the multi-layered core–shell structure was investigated for the synthesis of 1,2,4,5-tetrahydro-2,4-dioxobenzo[*b*][1,4]diazepine malononitriles *via* five-component reactions and also malonamide derivatives *via* pseudo six-component reactions at room temperature in ethanol. The examination of the two above-mentioned MCRs under sonication resulted in significant reduction in the reaction time. The recovery and reusability of the bionanocomposite were also checked successfully within 3 runs, and its FESEM and EDAX analysis demonstrated the stability of its structure.

## Conflicts of interest

There are no conflicts to declare.

## Supplementary Material

RA-012-D2RA00212D-s001
