# Supplementary material for: Metal-bio functionalized bismuthmagnetite [Fe3−xBixO4/SiO2@l-ArgEt3+I−/Zn(ii)]: a novel bionanocomposite for the synthesis of 1,2,4,5-tetrahydro-2,4-dioxobenzo[b][1,4]diazepine malononitriles and malonamides at room temperature and under sonication
Source: RSC Adv. 2022 Apr 1;12(17):10219–36. doi: 10.1039/d2ra00212d (PMC8972908; doi:10.1039/d2ra00212d)
Supplement: RA-012-D2RA00212D-s001 [file RA-012-D2RA00212D-s001.pdf]

Electronic Supplementary Material (ESI) for RSC Advances.
This journal is © The Royal Society of Chemistry 2022

Metal-bio functionalized bismuthmagnetite [$Fe_{3-x}Bi_xO_4/SiO_2$@*L*-$ArgEt_3^+I^-$/Zn(II)]: A novel bionanocomposite for the synthesis of 1,2,4,5-tetrahydro-2,4-dioxobenzo[*b*][1,4]diazepin malononitriles and malonamides at room temperature and under sonic waves

Fatemeh Molaei Yielzoleh, Kobra Nikoofar*

Department of Chemistry, Faculty of Physics and Chemistry, Alzahra University, Tehran, Iran

Tabel of content

S1. ^{1}H NMR (DMSO, *d_6*) spectra of 2-(2-(Cyclohexylamino)-2-(2,4-dioxo-4,5-dihydro-1*H*-benzo[*b*][1,4]diazepin-3(2*H*)-ylidene)-1-(4-hydroxy-3-methoxyphenyl)ethyl)malononitrile **(6e)**

S2. GC-MASS spectra of 2-(2-(Cyclohexylamino)-2-(2,4-dioxo-4,5-dihydro-1*H*-benzo[*b*][1,4]diazepin-3(2*H*)-ylidene)-1-(4-hydroxy-3-methoxyphenyl)ethyl)malononitrile **(6e)**

S3. ^{1}H NMR (DMSO, *d_6*) spectra of 2-(2-(Cyclohexylamino)-2-(2,4-dioxo-4,5-dihydro-1*H*-benzo[*b*][1,4]diazepin-3(2*H*)-ylidene)-1-(4-formylphenyl)ethyl)malononitrile **(6f)**

S4. GC-MASS spectra of 2-(2-(Cyclohexylamino)-2-(2,4-dioxo-4,5-dihydro-1*H*-benzo[*b*][1,4]diazepin-3(2*H*)-ylidene)-1-(4-formylphenyl)ethyl)malononitrile **(6f)**

S5. ^{1}H NMR (DMSO, *d_6*) spectra of *N^1,N^3*-bis(4-aminophenyl)-2-(3,3-dicyano-1-(cyclohexylamino)-2-(1*H*-indol-3-yl)propylidene)malonamide **(7f)**

S6. GC-MASS spectra of *N^1,N^3*-bis(4-aminophenyl)-2-(3,3-dicyano-1-(cyclohexylamino)-2-(1*H*-indol-3-yl)propylidene)malonamide **(7f)**

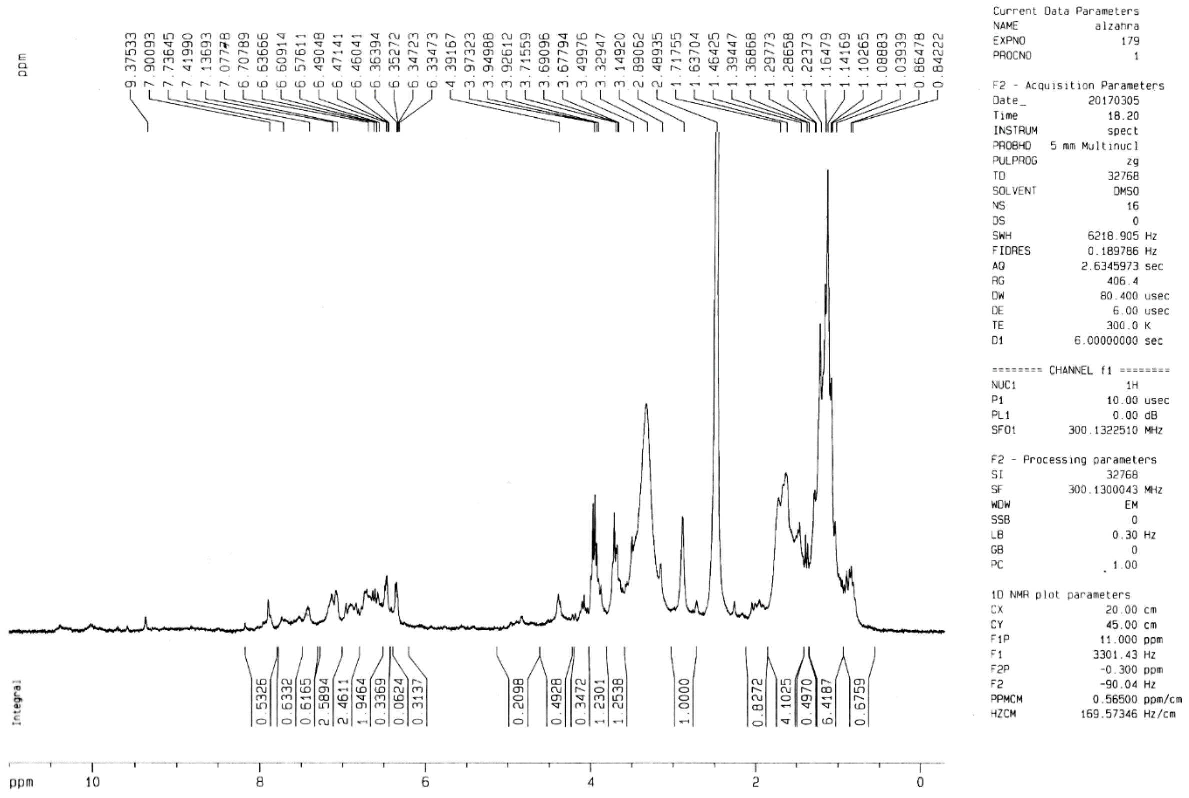

S1. ^{1}H NMR (DMSO, d_6) spectra of **(6e)**

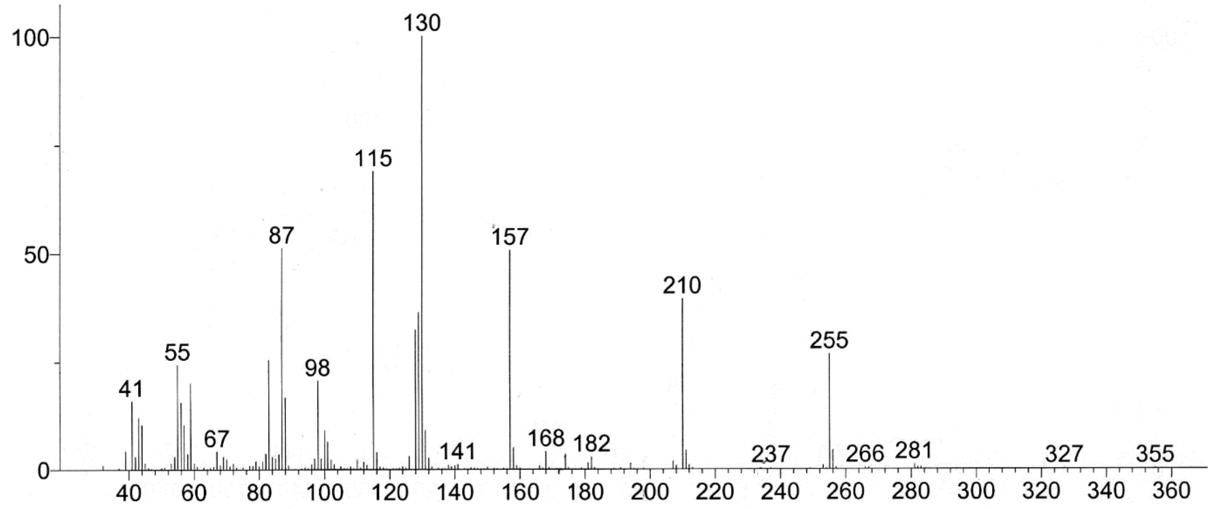

S2. GC-MASS spectra of **(6e)**

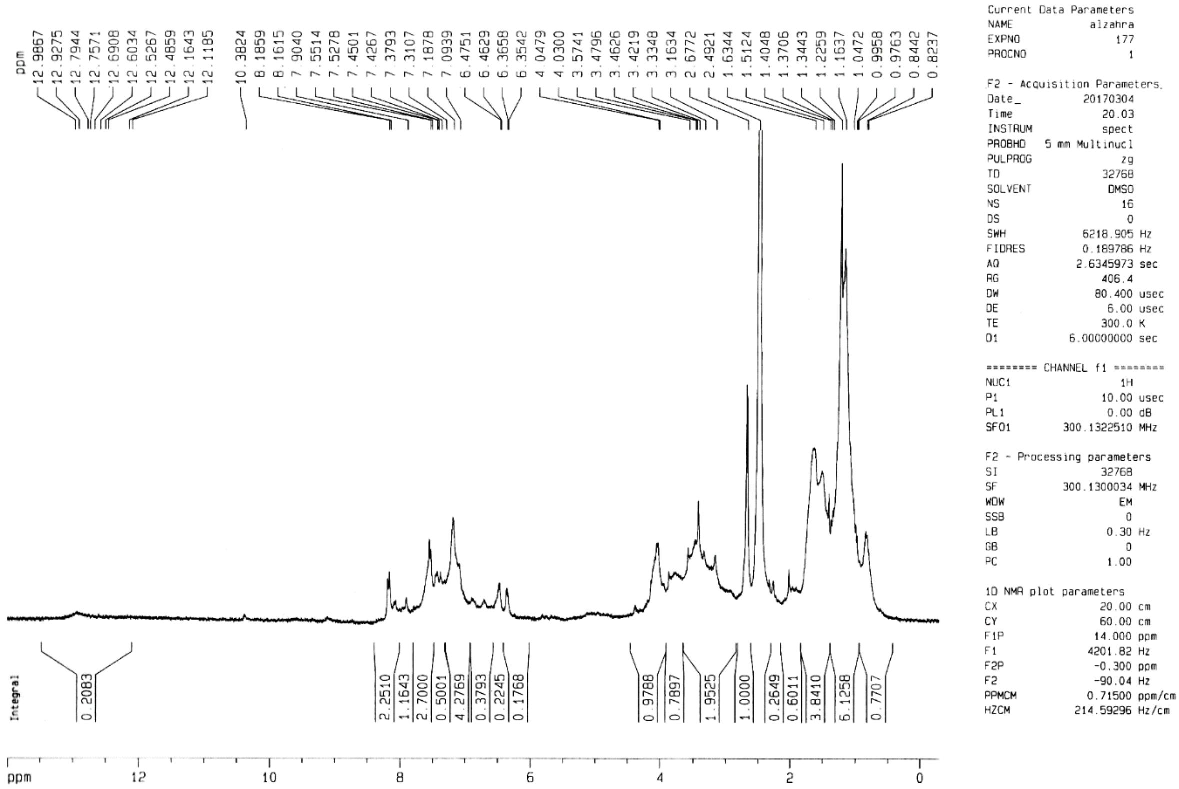

S3. ^{1}H NMR (DMSO, d_6) spectra of **(6f)**

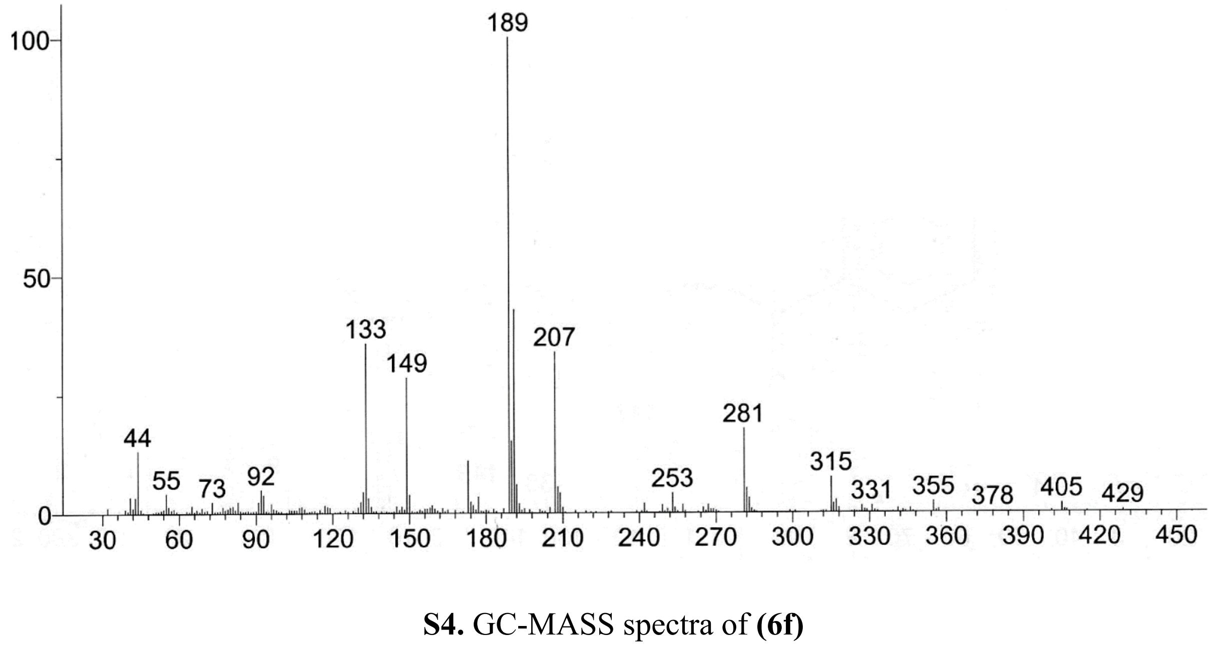

S4. GC-MASS spectra of **(6f)**

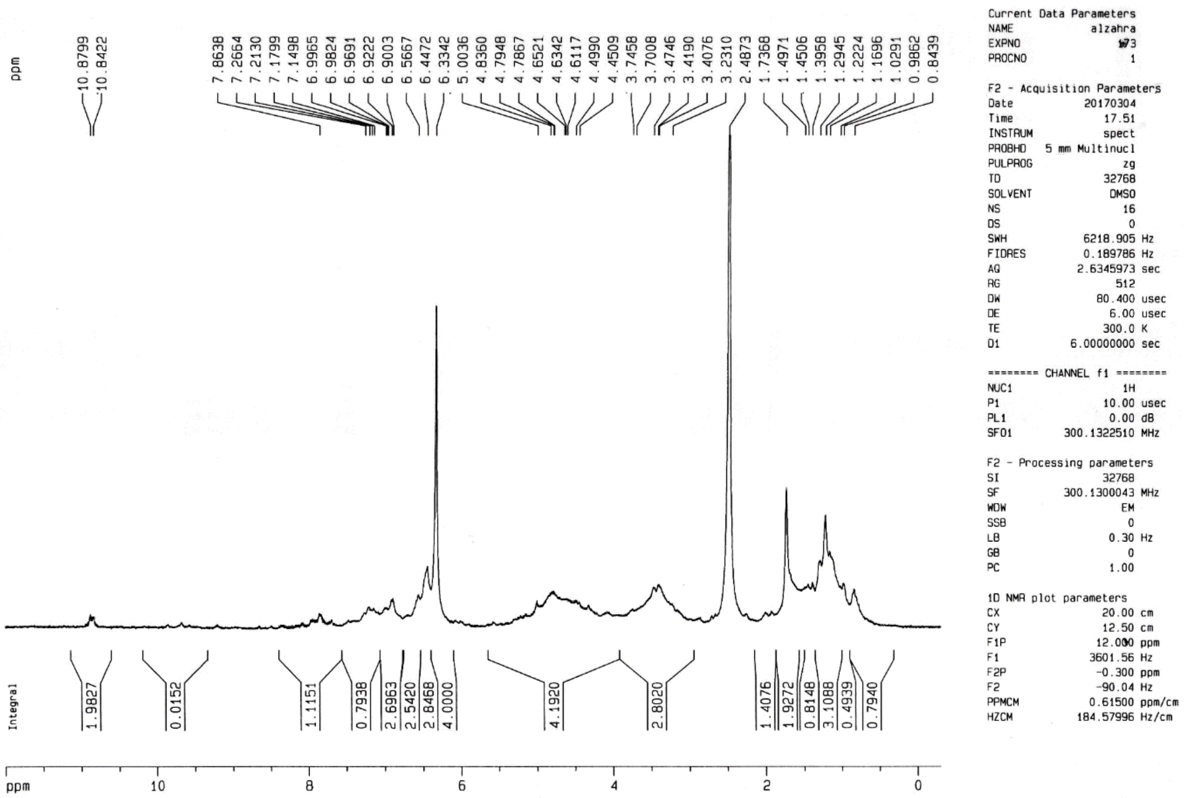

S5. ^{1}H NMR (DMSO, d_6) spectra of **(7f)**

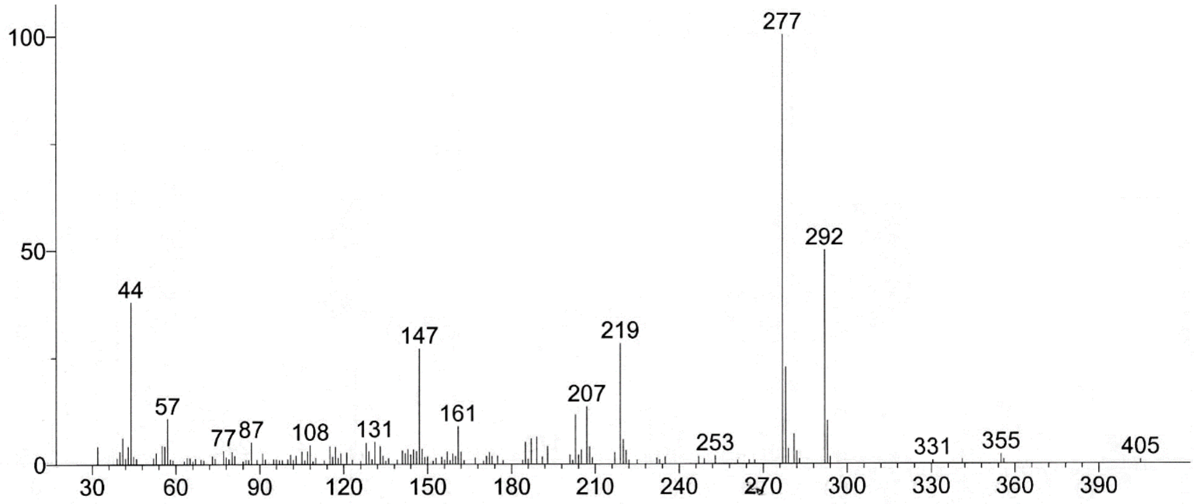

S6. GC-MASS spectra of **(7f)**
